# The role of junctophilin proteins in cellular function

**DOI:** 10.1152/physrev.00024.2021

**Published:** 2022-01-10

**Authors:** Stephan E. Lehnart, Xander H. T. Wehrens

**Affiliations:** ^1^Cellular Biophysics and Translational Cardiology Section, Heart Research Center Göttingen, University Medical Center Göttingen, Georg-August University Göttingen, Göttingen, Germany; ^2^Department of Cardiology and Pneumology, University Medical Center Göttingen, Georg-August University Göttingen, Göttingen, Germany; ^3^Cluster of Excellence “Multiscale Bioimaging: from Molecular Machines to Networks of Excitable Cells” (MBExC), University of Göttingen, Göttingen, Germany; ^4^DZHK (German Centre for Cardiovascular Research), partner site Göttingen, Germany; ^5^Cardiovascular Research Institute, Baylor College of Medicine, Houston, Texas; ^6^Department of Molecular Physiology and Biophysics, Baylor College of Medicine, Houston, Texas; ^7^Department of Medicine (Cardiology), Baylor College of Medicine, Houston, Texas; ^8^Department of Pediatrics (Cardiology), Baylor College of Medicine, Houston, Texas; ^9^Department of Neuroscience, Baylor College of Medicine, Houston, Texas; ^10^Center for Space Medicine, Baylor College of Medicine, Houston, Texas

**Keywords:** cardiomyopathy, excitation-contraction coupling, heart failure, junctophilins

## Abstract

Junctophilins (JPHs) comprise a family of structural proteins that connect the plasma membrane to intracellular organelles such as the endo/sarcoplasmic reticulum (ER/SR). Tethering of these membrane structures results in the formation of highly organized subcellular junctions that play important signaling roles in all excitable cell types. There are four JPH isoforms, expressed primarily in muscle and neuronal cell types. Each JPH protein consists of six membrane occupation and recognition nexus (MORN) motifs, a joining region connecting these to another set of two MORN motifs, a putative alpha-helical region, a divergent region exhibiting low homology between JPH isoforms, and a carboxy-terminal transmembrane region anchoring into the ER/SR membrane. JPH isoforms play essential roles in developing and maintaining subcellular membrane junctions. Conversely, inherited mutations in JPH2 cause hypertrophic or dilated cardiomyopathy, while trinucleotide expansions in the JPH3 gene cause Huntington Disease-Like 2. Loss of JPH1 protein levels can cause skeletal myopathy, while loss of cardiac JPH2 levels causes heart failure and atrial fibrillation, among other disease. This review will provide a comprehensive overview of the JPH gene family, phylogeny, and evolutionary analysis of JPH genes and other MORN domain proteins. JPH biogenesis, membrane tethering, and binding partners will be discussed, as well as functional roles of JPH isoforms in excitable cells. Finally, potential roles of JPH isoform deficits in human disease pathogenesis will be reviewed.

## Graphical abstract

**Figure:**
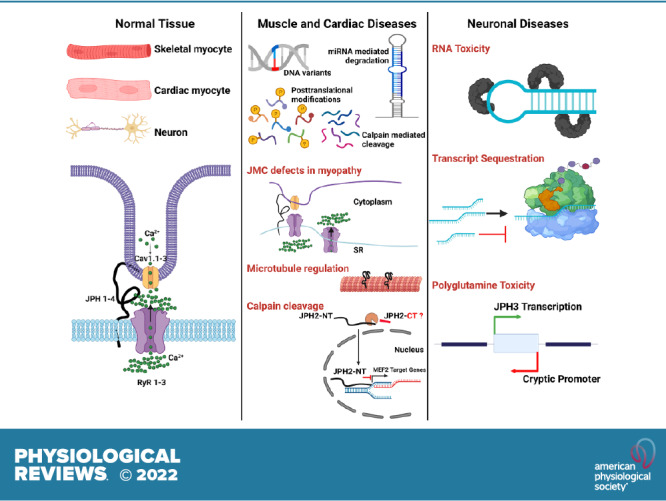

CLINICAL HIGHLIGHTS

1. Junctophilins (JPHs) play an essential role in excitable cell types such as striated muscle cells and neurons. They provide structural integrity to the junctional membrane complexes between the plasma membrane and endo/sarcoplasmic reticulum. Altered junctophilin expression or function impacts intracellular calcium handling and/or ion channel function thereby affecting cellular excitability.
2. Inherited variants in the *JPH1* gene have been proposed to play a disease-modifier role in a rare form of Charcot-Marie-Tooth disease caused by inherited variants in the ganglioside-induced differentiation-associated protein 1 (*GDAP1*) gene.
3. Inherited variants in the *JPH2* gene cause hypertrophic cardiomyopathy, a genetic disorder characterized by left ventricular hypertrophy and an increased risk of cardiac arrhythmias, and in rare cases dilated cardiomyopathy. *JPH2* variants cause defective intracellular calcium handling, resulting in cellular hypertrophy and an increased propensity toward arrhythmias.
4. Trinucleotide repeat expansions in the *JPH3* gene can cause Huntington Disease-Like 2, a neurodegenerative disease characterized by movement, psychiatric, and cognitive abnormalities. The disease etiology has been attributed to cellular toxicity of RNA foci, a loss of JPH3 protein expression due to disrupted translation, or polyglutamine toxicity resulting from transcription of a cryptic gene on the antisense strand.
5. Reduced JPH2 protein levels have been observed in patients with non-genetic forms of heart failure and cardiomyopathy. Loss of JPH2 causes a reduction in transverse tubule invaginations and junctional membrane complexes, which impairs excitation-contraction coupling and leads to contractile dysfunction.

## 1. INTRODUCTION

### 1.1. Junctional Membrane Complexes

Junctional membrane complexes (JMCs) are a common feature among all excitable cell types (1, 2). These specialized subcellular domains, containing discontinuous membrane junctions of plasma membrane and endoplasmic/sarcoplasmic reticulum (ER/SR), mediate cross talk between the cell surface and intracellular ion channels (3, 4). In striated muscle cells, JMCs additionally couple sarcolemmal invaginations known as transverse (T)-tubules and the SR (5). Within the 12- to 15-nm cleft of the JMCs (6), clusters of voltage-gated L-type calcium (Ca^2+^) channels (LTCC) densely clustered on the T-tubular membrane and intracellular Ca^2+^ release channels known as ryanodine receptors (RyR) are arranged such that they communicate efficiently within the JMC subspace.

In skeletal muscle, depolarization of LTCCs induces a conformational change that, by means of direct physical interactions with RyR1 channels, initiates Ca^2+^ release through a mechanism termed depolarization-induced Ca^2+^ release (7). In cardiac muscle, Ca^2+^ influx through LTCC leads to the release of Ca^2+^ via RyR2, a process known as Ca^2+^-induced Ca^2+^ release (8, 9). Hence, in both major types of striated muscle, the release of Ca^2+^ from the SR leads to increased levels of cytosolic Ca^2+^ that, in turn, activate actin-myosin cross-bridge formation and muscle cell contraction. These principally different processes of excitation-contraction (E-C) coupling are critical for coupling of cellular depolarization and contractile function of striated muscle cells (10). Interestingly, junctions between the plasma membrane and ER exist also in neurons, where they facilitate communication between cell surface and intracellular ion channels that modulate excitability and synaptic plasticity (11). In addition, JMCs in sensory neurons may also mediate proinflammatory G protein-coupled receptor signaling that mediates the generation of inflammatory pain (12).

### 1.2. Discovery of Junctophilins

**FIGURE 1.**
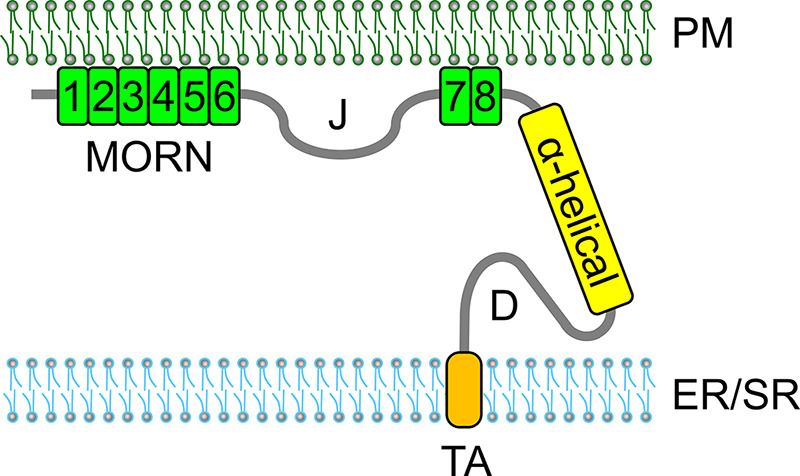
Predicted subcellular organization of junctophilin (JPH) within the junctional membrane complex consisting of the plasma membrane (PM) and endoplasmic/sarcoplasmic reticulum (ER/SR). JPH2 attaches to the PM by means of 2 sets of membrane occupation and recognition nexus (MORN) domains (green), connected by a junctional domain (J). The divergent (D) region with low homology among JPH isoforms. The COOH-terminal tail of JPH2 anchors into the endo/sarcoplasmic reticulum (ER/SR) membrane using a transmembrane domain, TA, tail anchor.

In 2000, Takeshima et al. (2) identified the first structural proteins involved in JMCs in rabbit skeletal muscle using a monoclonal antibody screen. In particular, the junctophilin-1 (JPH1) sequence was identified as the tether localized within triadic JMCs situated between T-tubule membrane invaginations and the SR in skeletal muscle cryosections (2). Subsequently, four junctophilin protein isoforms, JPH1, JPH2, JPH3, and JPH4, were reported with a length of 661, 696, 748, and 628 amino acids (aa), respectively (13, 14). All isoforms share distinct structural features deduced from their hydropathicity profiles and predicted secondary structures, as well as sequence homology with other proteins (13). Functionally important, the NH_2_ terminus contains six membrane occupation and recognition nexus (MORN) motifs, a joining region connecting this region to another domain containing two MORN motifs, a putative α-helical region, a divergent region exhibiting low homology between JPH isoforms, and a COOH-terminal transmembrane region, also known as the tail anchor, spanning the ER/SR membrane (FIGURE 1) (13).

Since the discovery of junctophilins in 2000, research on this family of proteins has revealed vastly different cellular roles in healthy and diseased tissues. In this review, we will discuss the genetic origin and organization of *JPH* genes, as well as inherited variants associated with human disease development. The biogenesis, subcellular organization, and binding partners of JPH isoforms in different excitable cell types will be discussed and compared between distinct cell types. Finally, we will discuss mechanisms by which JPH defects or altered expression levels can contribute to the development of various diseases.

## 2. JUNCTOPHILIN GENE FAMILY

### 2.1. Genomic Location and Organization of JPH Genes

#### 2.1.1. Genomic location of JPH genes.

**FIGURE 2.**
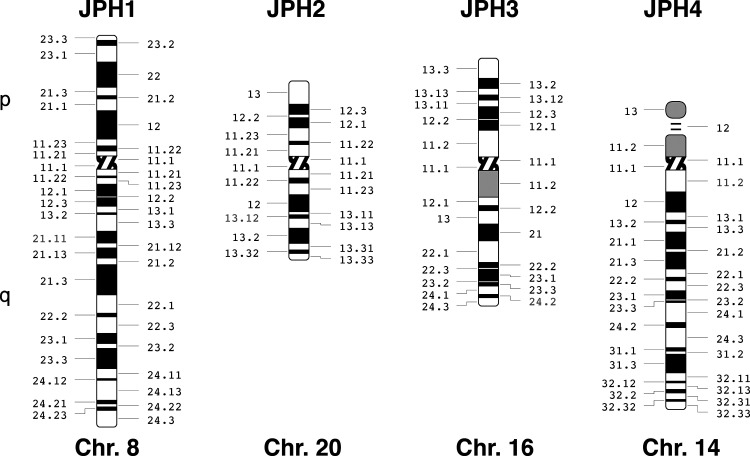
Localization of junctophilin (JPH) genes on chromosomes. Ideograms of chromosomes (Chr.) 8, 20, 16, and 14 (p and q regions), showing the *JPH1*, *JPH2*, *JPH3*, and *JPH4* gene locations, respectively. Cytogenic bands are showing in accordance with the International System for Cytogenetic Nomenclature (15). Idiograms were generated using files posted on the website of the Department of Pathology of the University of Washington (16).

Nishi et al. (13) isolated the human *JPH1* and *JPH2* genes by screening a genomic DNA library and isolated *JPH3* from a brain cDNA library. The first exon of *JPH3* could not be isolated from several genomic DNA libraries, presumably because the *JPH3* gene maps to the terminal region of a chromosome. Genomic mapping revealed that *JPH* genes do not cluster on the human genome (13). The *JPH1* gene is located on the long arm of chromosome 8 at cytogenetic band 8q21.11, according to the HUGO Gene Nomenclature Committee and Ensembl (FIGURE 2). The gene spans ∼86,841 bases and is located on the minus strand of chromosome 8. The *JPH2* gene was originally cloned from genomic DNA segments derived from the human chromosome 20q12 region (13). Subsequent studies revealed that the JPH2 gene is located on the long arm of chromosome 20 at the 20q13.12 band (FIGURE 2). The gene spans ∼81,357 bases and is located on the minus strand of the chromosome.

Nishi et al. (13) initially mapped the human *JPH3* gene to 16q23-q24 using fluorescence in situ hybridization (FISH). Subsequently, Holmes et al. (17) localized the *JPH3* gene more precisely to 16q24.3 on the basis of sequence data provided by the Human Genome Project (FIGURE 2). The gene spans 96,322 bases and is on the plus strand at the distal end of the long arm of chromosome 16. Finally, the *JPH4* gene is located on the minus strand of the long arm of chromosome 14 at cytogenetic band 14q11.2 (FIGURE 2). The *JPH4* gene spans merely 10,753 bases due to shorter intronic regions, whereas all other *JPH* genes include long intronic sequences that have unusually large sizes.

#### 2.1.2. Genomic organization of JPH genes.

**FIGURE 3.**
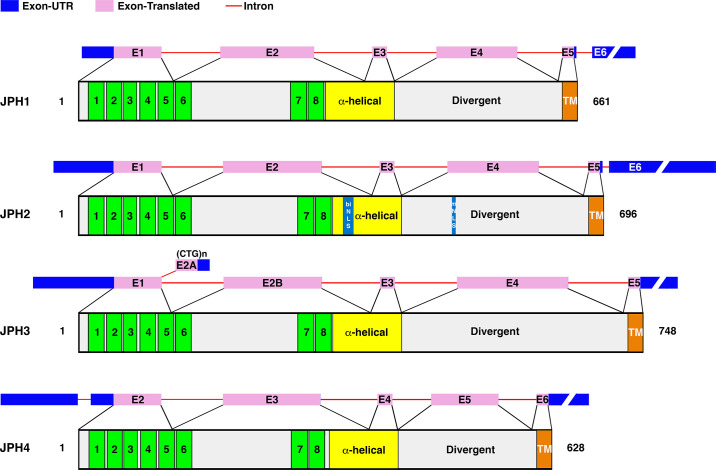
Gene and protein diagram of junctophilin (JPH) isoforms. Diagram shows gene diagram including exons and introns in correlation with the protein topology for the 4 JPH isoforms expressed in humans. The membrane occupation and recognition nexus (MORN) domains are shown in green, while the other domains are marked. The exons (E) are shown in pink. JPH, junctophilin; biNLS, biphasic nuclear localization signal; mNLS, monopartite nuclear localization signal; UTR, untranslated region; TM, transmembrane domain.

Experimental evidence suggests that the human *JPH1–JPH3* genes each contain five exons separated by four introns (FIGURE 3) (13). In addition, there are NH_2_- and COOH-terminal untranslated regions. Among the human *JPH* isoform genes, the exon-intron junctions locate to identical positions in their aligned amino acid sequences. In *Caenorhabditis elegans*, the single *JPH* gene contains 10 exons and 10 exon-intron junctions. The first intron of the human genes and the predicted second intron of the nematode gene separate the respective protein-coding sequences at an identical aligned position. However, other exon-intron boundaries are localized at different aligned positions between the *Homo sapiens* and *C. elegans* genes (13). Similar differences in gene organization among diverse organisms are known for many genes. This supports the model that introns have been inserted into preexisting genes during eukaryotic evolution (18, 19).

Various sequence annotation viewers including Entrez, Ensembl, and AceView suggest that human *JPH* genes can be alternatively spliced (20). For *JPH1*, three mRNA variants (4,489 bp, 3,997 bp, and 1,880 bp) have been observed in sequencing databases (as per AceView). However, there is no definitive experimental evidence at the present time. The primary 4,489-bp mRNA leads to the formation of the canonical 661-aa JPH1 protein with a predicted molecular mass of 71 kDa. It is currently unknown if the *JPH2* gene undergoes alternative splicing, although three alternative mRNA sequences (2,487 bp, 1,759 bp, and 839 bp) have been reported in the AceView database. The primary mRNA (2,487 bp) leads to the formation of the 696-aa JPH2 protein, which has a predicted molecular mass of 74 kDa. One of the variants uses an alternate 3′-terminal exon, resulting in a shorter isoform.

There is convincing experimental evidence that the *JPH3* gene is alternatively spliced and that trinucleotide repeats in an alternatively spliced exon cause a neuronal disease (see also sect. 5.3) (21). Eight different mRNA variants have been reported in the AceView database (4,002 bp, 2,407 bp, 4,022 bp, 1,800 bp, 451 bp, 619 bp, 541 bp, and 473 bp). The primary 4,002-bp mRNA encodes the full-length JPH3 protein consisting of 748 aa with a predicted molecular mass of 81 kDa. The CTG trinucleotide repeats are inserted within the alternatively spliced exon 2a, the length of which varies depending on the repeat number (FIGURE 3). Finally, *JPH4* is also predicted to undergo alternative splicing although it is unclear from AceView how many mRNA variants exist due to a joint listing with another gene (adaptor-related protein complex 1, gamma 2 subunit) that is located on the same chromosomal locus (22).

#### 2.1.3. JPH gene expression patterns.

RNA blot hybridization revealed that the tissue-specific expression patterns of *JPH* genes in humans are essentially the same as those in mice (13). Subsequent RNA sequencing of total RNA from 20 human tissues and serial analysis of gene expression summarized in GeneCards (23) revealed that *JPH1* mRNA expression is most abundant in skeletal muscle, followed by brain, heart, lung, prostate, thymus, and thyroid. In human tissues, *JPH2* mRNA is expressed primarily in skeletal and cardiac muscle, uterus, prostate, stomach, and small intestine. The expression of *JPH3* mRNA is more limited to the brain (including cerebellum), and low expression was seen in the thymus, kidney, and adrenal gland. Finally, *JPH4* mRNA expression showed a similar pattern of high expression in brain tissue, with lower levels in prostate, uterus, and the adrenal gland. *JPH4* was also detected in T-lymphocytes (24).

### 2.2. Phylogeny and Evolutionary Conservation of JPH Isoforms

#### 2.2.1. Phylogenetic analysis of JPH isoforms.

Phylogenetic trees are routinely generated in biology to present and interpret the evolutionary relationships of species (25). A phylogenetic tree can also provide an estimate of the relationships among DNA or protein sequences themselves without regard of the host species, inferring the functions of genes or proteins that have not been studied experimentally (26). While prior phylogenic studies on JPH have been reported (27–29), we performed a new analysis taking into consideration all currently available sequencing information. Within this context, Mackrill and Shiels (29) recently published a comprehensive analysis of several key proteins relevant for excitation-contraction coupling including junctophilins.

The phylogenetic tree was created using the protein sequences of 57 JPH isoforms retrieved from Ensembl following a protein BLAST query to identify more distant, less studied species. Twenty-two different species were selected based on different times of the last common ancestor according to OneZoom (TABLE 1) (31). TimeTree was used to obtain the estimates of evolutionary distances between humans and selected species (30). The JPH isoforms were found to be present and conserved across phyla of the animal kingdom. There were 32 mammalian JPH sequences, 17 from lower vertebrates, and 8 from invertebrates. The JPH protein sequences ranged in size from 391 to 1054 aa. The insect sequence (*Drosophila melanogaster*) consists of 1054 aa due to an extra segment in the divergent region, which may have been the result of an arthropod-specific divergence or duplication. Similar results have been reported before for the honey bee (*Apis mellifera*), wasp (*Nasonia vitripennis*), and other *Drosophila* species (27).

**Table 1.** Junctophilin isoforms included in phylogenetic tree analysis

Scientific Name	Common Name	Isoforms	Estimated Evolutionary Distance, MYA
*Homo sapiens*	Human	1, 2, 3, 4	0
*Macaca mulatta*	Rhesus monkey	1, 2, 3, 4	29
*Callithrix jacchus*	Common marmoset	1, 2, 3, 4	43
*Mus musculus*	House mouse	1, 2, 3, 4	90
*Sus scrofa*	Wild boar/domestic pig	1, 2, 3, 4	96
*Chrysochloris asiatica*	Cape golden mole	1, 2, 3, 4	105
*Sarcophilus harrisii*	Tasmanian devil	1, 2, 3, 4	159
*Ornithorhynchus anatinus*	Platypus	1, 2, 3, 4	177
*Gallus gallus*	Red junglefowl/domestic chicken	1, 2, 3	312
*Xenopus laevis*	African clawed frog	1, 2, 3, 4	352
*Latimeria chalumnae*	West Indian Ocean coelacanth	1, 2, 3	413
*Danio rerio*	Zebrafish	1, 2, 3	435
*Scyliorhinus canicula*	Small-spotted catshark	1, 2, 3	473
*Petromyzon marinus*	Sea lamprey		615
*Styela clava*	Stalked sea squirt		676
*Branchiostoma floridae*	Florida lancelet		684
*Patiria miniata*	Bat star		684
*Drosophila melanogaster*	Common fruit fly		797
*Caenorhabditis elegans*	Roundworm		797
*Nematostella vectensis*	Starlet sea anemone		824
*Trichoplax adhaerens*	Trichoplax		948
*Amphimedon queenslandica*	Amphimedon queenslandica		952

The estimated evolutionary distance was obtained from the TimeTree database (30). MYA, million years ago.

**FIGURE 4.**
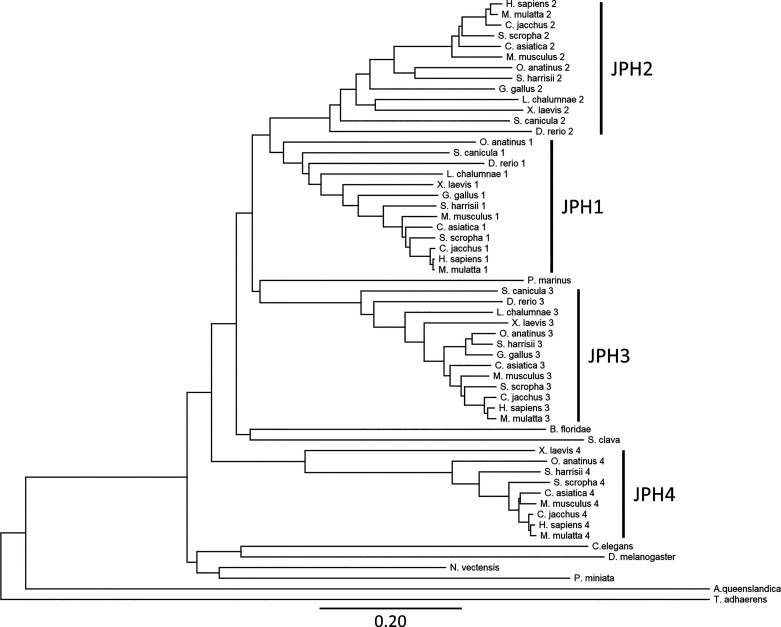
Phylogenetic tree of junctophilin (JPH) isoforms. The phylogenetic tree was created using 57 JPH isoforms from 22 different species that were selected with different times of the last common ancestor. Protein sequences were aligned using MUSCLE in Mega-X using default settings, a distance matrix was generated, and the phylogenetic tree was generated using the neighbor joining method. Clades containing each of the isoforms are indicated. The scale bar represents the length of each branch as the average number of amino acid substitutions per site (per Mega-X).

The sequences were aligned using MUSCLE in Mega-X using default settings, a distance matrix was generated, and a phylogenetic tree was generated using the neighbor joining method (FIGURE 4) (32). This method was chosen because it grouped the different JPH isoforms and clades the best. The phylogenetic tree shows that JPH emerged in *Amphimedon queenslandica*, a sponge of the phylum Porifera. These very basic Metazoa (animals) are multicellular organisms that lack a nervous system but do have myocytes that cause parts of the animal to contract (33). Other studies have suggested that *Salpingoeca rossetta*, which belongs to the choanoflagellate, is the most basal organism possessing a JPH homologue (29). The choanoflagellates are a group of unicellular and colonial flagellate eukaryotes considered to be the closest living relatives of the animals. Interestingly, JPH homologues were not detected in another choanoflagellate species, *Monsiga brevicollis* (29). Moreover, JPH was not found in filasterea, another sister group to animals represented by the organism *Capsaspora owczarzaki* (29, 34).

The ancestral invertebrates and the disputed vertebrate *Petromyzon marinus* from the Hypercarfia class of Chordates all only have a single JPH isoform (also referred to as JPHa). This group includes several phyla including the Porifera, Placozoa (*Trichoplax adhaerens*), Cnidaria (*Nematostella vectensis*), Nematoda (*C. elegans*), Arthropoda (*D. melanogaster*), Echinodermata (*Patiria miniata*), and the Chordata (*Branchiostoma floridae, Styela clava,* and *Perkinsus marinus*). The fact that the *N. vectensis* sequence is not grouped with the other nonbilaterals (*Amphimedon queenslandica* and *T. adhaerens*) is consistent with it being more closely related to the bilaterals, combined with the rapid divergence and gene loss of arthropod and nematode genomes compared with the Cnidarias (35). The presence of a JPH homologue has only been verified experimentally for two of these species, the *C. elegans* and *D. melanogaster* (36, 37). The single JPH isoform appears to play roles in skeletal muscle, the heart, and neurons, suggesting that the single JPH protein can be functionally equivalent to its four mammalian paralogues (34, 37).

The number of JPH genes increased from a single gene to three to four genes after chordates branched from other deuterostomes, around the time vertebrate arose (i.e., the *Scyliorhinus canicula* has three JPH isoforms; FIGURE 4). The ancestor to the vertebrate *JPH* gene family underwent two separate gene duplication events, most likely first giving rise to a muscle and a neuronal isoform. The JPH1–2 clades then separated from the common muscle ancestor, whereas the JPH3–JPH4 clades diverged from the neuronal ancestor (27). While these events most likely occurred during the Cambrian explosion, a 13- to 25-million-year period during, which most modern metazoan phyla emerged (38), the exact timing of the serial gene duplications could not be established due to the lack of JPH sequences from organisms that emerged during specific Cambrian phases (39). It appears that one of the four JPH genes was lost in certain lineages, including the classes of chondrichthyes (cartilaginous fishes), osteichthyes (bony fishes), and aves (birds) (see TABLE 1 and FIGURE 4) (29). On the other hand, it has been suggested that teleost fish genomes contain a duplicated pair of JPH1 genes, in addition to JPH2 and JPH3 genes (29). It is believed that the JPH1 duplication may have occurred during a teleost-specific third round of whole genome duplication, followed by loss of one of the copies of JPH2 and JPH3 (40).

FIGURE 4 shows that the JPH sequences from vertebrates are distinctly grouped into four clades (JPH1–4). Within each clade, the mammalian and nonmammalian proteins are organized in a manner consistent with the evolution of the species. The JPH isoforms are highly conserved across vertebrate species, suggesting that there has been significant evolutionary pressure to remain relatively unchanged, despite the fact that four distinct isoforms were generated by duplication events early among vertebrates. The JPH4 isoform exhibits a longer branch length relative to the other JPH isoforms, suggesting that it has undergone the most sequence changes. This may imply that JPH4 role is more of a complementary one in the brain, compared with the more specific and essential roles played by JPH3 in the brain and JPH1–2 in striated muscle (27).

#### 2.2.2. Evolutionary trace of JPH isoforms.

**FIGURE 5.**
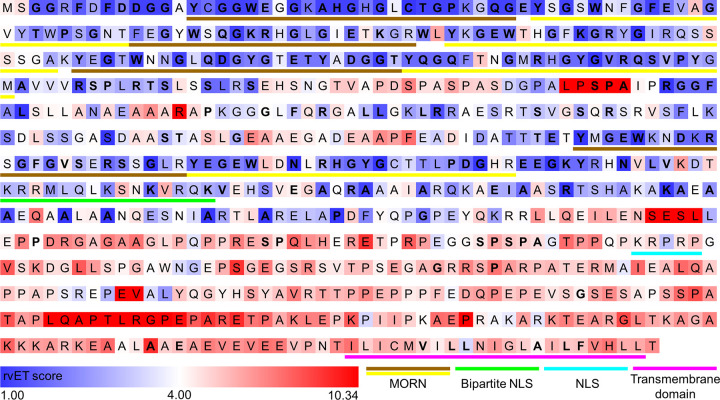
Evolutionary trace mapping of human junctophilin 2 (JPH2). Human JPH isoforms were aligned using the MUSCLE function in Mega-X using default settings. The universal evolutionary trace was generated using the Evolutionary Trace viewer created by Dr. O. Lichtarge (44). The human JPH2 sequence is shown as the reference sequence; the output was color coded based on the evolutionary trace scores (rvET) from the RANKS files. Lower scores (blue shaded squares) represent higher importance; higher scores represent a lower importance of amino acids (red shaded squares). Bolded amino acids are conserved across isoforms. Functional domains are underlined. MORN, membrane occupation and recognition nexus; NLS, nuclear localization signal.

The evolutionary trace is the most validated approach to identify protein functional determinants (41, 42). The concept behind this approach is that protein structures descending from a common ancestor are remarkably similar with very minor backbone deviations (43) and functionally important residues undergo fewer mutations than less important amino acids (41). This predictive computational method scans a multiple sequence alignment for residue variations that correlate with major evolutionary divergences (42). We generated an evolutionary trace for junctophilins by aligning human JPH isoforms using the MUSCLE function in Mega-X and using the Evolutionary Trace viewer created by Dr. O. Lichtarge to display the results (44). The output was color coded based on the evolutionary trace scores (rvET) from the RANKS files; residues in blue are more important, while those in red are less important for protein function (FIGURE 5).

The evolutionary trace (ET) revealed that the eight MORN domains are among the most important and highest conserved regions of JPH isoforms. Whereas the MORN motifs exhibit the highest degree of conservation among different JPH domains (∼80%), the overall identity between the JPH1 and JPH4 isoforms (∼40%) was lower (27). This suggests that the gene duplication and subsequent divergence of different JPH isoforms may have evolved for specific functions in different excitable tissues. The bipartite nuclear localization signal (bNLS) was also well conserved, whereas the conservation of the second more COOH-terminal monopartite NLS (mNLS) was lower. The importance of these functional domains is discussed in sect. 6.4.4. Surprisingly, the COOH-terminal region containing the transmembrane domain showed a lower ET score. This lower score might be caused by the predicted absence of a transmembrane topology in species such as *T. adhaerens*, *N. vectensis*, and *Hydrocotyle vulgaris* (29) using different algorithms such as Phobius (45) and THHM (46). This prediction could be incorrect as a result of poor sequence quality or annotation, leading to artifactually truncated hypothetical proteins. Alternatively, these basal metazoan JPH homologues may lack the transmembrane domain that anchors JPH into the ER domain but still regulate the voltage-gated calcium channel, consistent with studies on an engineered COOH-terminally truncated form of mammalian JPH1 that still inhibits gating of Cav1.1 Ca^2+^ channels (47).

#### 2.2.3. Evolutionary conservation of MORN domains in JPH.

**FIGURE 6.**
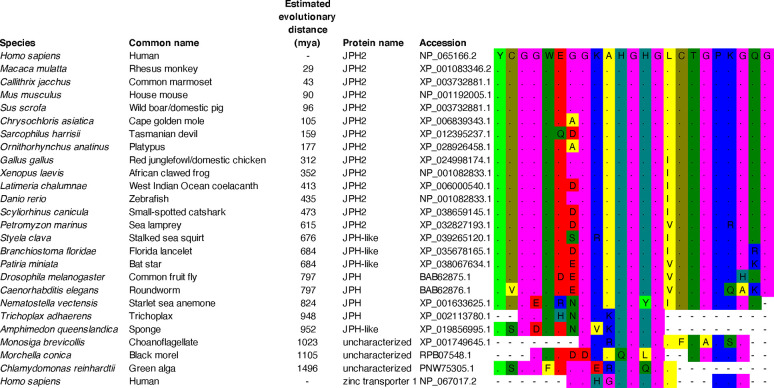
Evolutionary conservation of the junctophilin-2 (JHP2) membrane occupation and recognition nexus (MORN)-1 domain. Homologues of the *Homo sapiens* JPH2 protein were obtained using protein BLAST (see accession numbers). The selected top hit from each species with different evolutionary distances from humans were aligned using the MUSCLE feature in Mega-X. A distance matrix of the aligned sequences was generated using default settings. Estimated evolutionary distance was obtained from TimeTree (30). The colors represent the amino acid types; mya, millions of years ago.

The domain with the single highest ET score is the first MORN domain (FIGURE 5). A protein BLAST search of this 23-aa domain revealed a high degree of conservation across species (FIGURE 6). The sequence is identical in most mammals but a little different in the more distantly related marsupials. Similar to the phylogenetic analysis of the full-length JPH isoforms, this MORN domain was present in JPH homologues in species as distantly related as the *A. queenslandica*, a sponge of the phylum Porifera. Interestingly, key parts of the MORN domain were also found in homologous, still uncharacterized proteins in *Monosiga brevicollis*, a member of the choanoflagellates. These small unicellular eukaryotes from the Protista Kingdom are comprised of both marine and freshwater species. According to current molecular phylogeny theories, choanoflagellates are the closest unicellular relative of metazoans (48). The BLAST search also revealed homology with another uncharacterized protein in *Morchella conica*, also known as the black morel, from the Fungi kingdom (49). In addition, a homologous protein was found in *Chlamydomonas reinhardtii*, a single-cell green alga with a diameter of ∼10 μm that swims with two flagella. This alga is from the Plantae kingdom and the most distantly related to the Animalia kingdom within the Eukaryota Superkingdom (50). Finally, this particular analysis did not reveal any homologous proteins from the Prokaryota Superkingdom.

**FIGURE 7.**
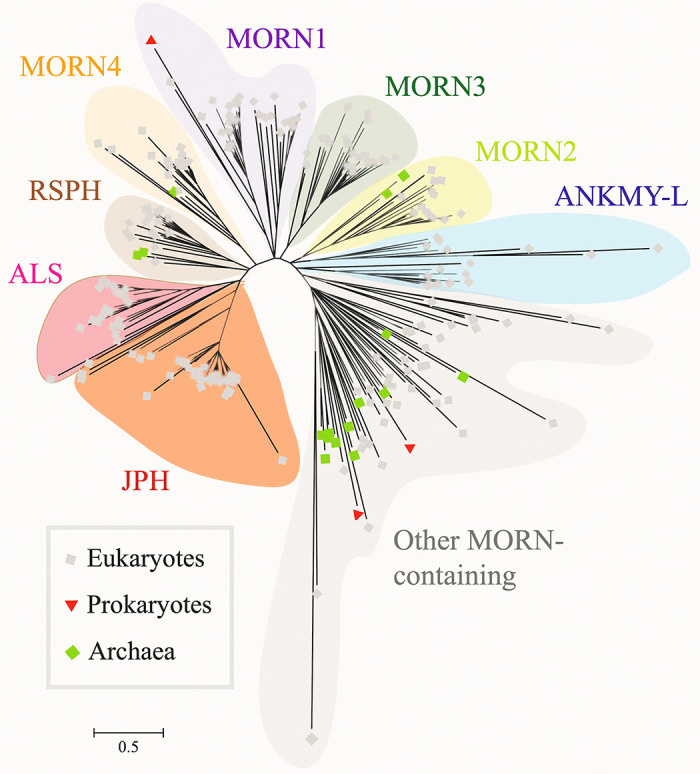
Phylogenetic tree of membrane occupation and recognition nexus (MORN) repeat containing protein families. The phylogenetic tree was constructed using 238 amino acid sequences from a range of proteins found in eukaryotes, bacteria, and archaea. The clustering of this tree revealed 8 distinct families of protein containing MORN repeat-containing proteins: the junctophilins (JPH), alsins (ALS), radial spoke-head homology (RSPH) proteins, four MORN-containing families (MORN1-MORN4), and an ankyrin repeat and MYND domain-containing protein 1-like (ANKMY-L) family, along with another less clearly defined group of proteins. The tree is to scale, with branch lengths corresponding to amino acid substitutions per site, calculated using the average pathway method. Reproduced with permission from Mackrill and Shiels (29).

MORN domains have been found in other proteins in the human genome. Human zinc transporter 1 has the closest alignment with the junctophilin MORN-1 domain. This is notable because this protein also interacts with the L-type Ca^2+^ channel and inhibits its function (51). In addition, MORN domains have been previously reported in phosphatidylinositol-4-phosphate 5-kinases (PIP5Ks) and histone-lysine *N*-methyl-transferases (27). Mackrill and Shiels (29) recently identified other families of proteins containing MORN domains, including the families of MORN-repeat proteins (MORN1–4), 2-isopropylmalate synthases, ankyrin repeat and MYND domain-containing (ANKMY) proteins, radial spoke-head (RSPH) proteins, and alsin-like (ALS) proteins (FIGURE 7). They also identified candidate MORN superfamily members in the genomes of the viruses *Pandoravirus dulcis* and of *Bodo saltans* virus. According to the Simple Modular Architecture Research Tool (SMART) database (52, 53), there are 17,959 proteins with MORN domains from species from both the Eukaryota and Prokaryota Superkingdoms, as well as Viruses and undefined Kingdoms. According to this database, 55 *H. sapiens* proteins contain a total of 229 MORN domains.

It is anticipated that the MORN repeats in the protein families mentioned above interact with lipids, in particular phospholipids (29). As is the case for JPHs, certain MORN domain proteins play additional roles in determining the subcellular distribution and stability of protein complexes. For example, MORN4 family members can act as adaptors that tether class III myosin motor proteins to membranes (54). The RSPH proteins in eukaryotes are located in the central pair of microtubules of cilia or flagellae, where they regulate force production via interactions with the motor protein dynein (55). The phylogenetic tree of MORN domain-containing proteins shows that ALS proteins are most closely related to JPHs (FIGURE 7). In humans, the ALS2 (Alsin Rho guanine nucleotide exchange factor) gene is mutated in amyotrophic lateral sclerosis-2 (56). ALS2 plays a role in vesicle-mediated transport and regulates endocytosis by activation of the small G-protein Rab5, implying that the MORN domain plays a role in cellular motility and trafficking (57). Finally, a primary difference between JPH proteins and all other MORN repeat proteins is that the former possesses a transmembrane segment, crucial for localizing it to intracellular organelles.

## 3. JUNCTOPHILIN BIOGENESIS AND MOLECULAR FUNCTIONS

### 3.1. Junctophilin Biogenesis

#### 3.1.1. Biogenesis of tail-anchored proteins.

Proteins destined for membrane organelles contain short signal sequences in their transmembrane domains, which are recognized cotranslationally by the signal recognition particle. In contrast, tail-anchored (TA) proteins such as JPH1–4 and phospholamban residing in the ER membrane represent a specific class of membrane proteins characterized by a single transmembrane domain close to the COOH terminus [reviewed in Kutay et al. (58)]. In addition to the relatively thin ER membrane, the TA protein family populates physically thicker organelle membranes with over >300 members in humans, >50 in yeast, and >500 in plants (59). Thus the need to target JPH1–4 to the ER membranes is part of a fundamental biological process that supports correct subcellular compartmentalization.

**FIGURE 8.**
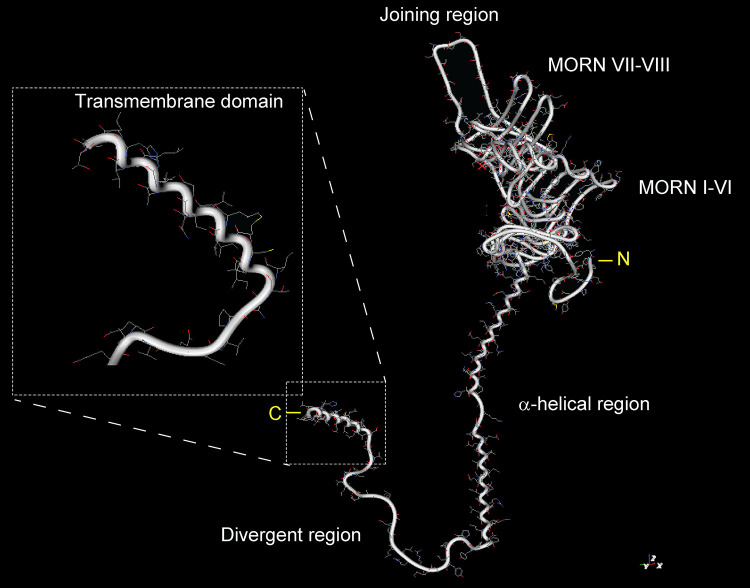
Junctophilin 2 (JPH2) 3-dimensional structural prediction model. JPH2 template-based tertiary structure modeling by RaptorX visualizing the backbone fold and atomic structure. Labels indicate major JPH2 domains and regions. *Inset*: magnification (dashed boxes) visualizing the COOH-terminal (C) transmembrane domain (TMD) α-helix of the JPH2 tail anchor in maximal side projection. N, NH_2_ terminal. Modified JPH2 model based on the same protein sequence from Gross et al. (60) using Molekel software. The color code for the atom structure is red for oxygen (O), blue for nitrogen (N), yellow for sulfur (S), and gray for carbon (C).

**FIGURE 9.**
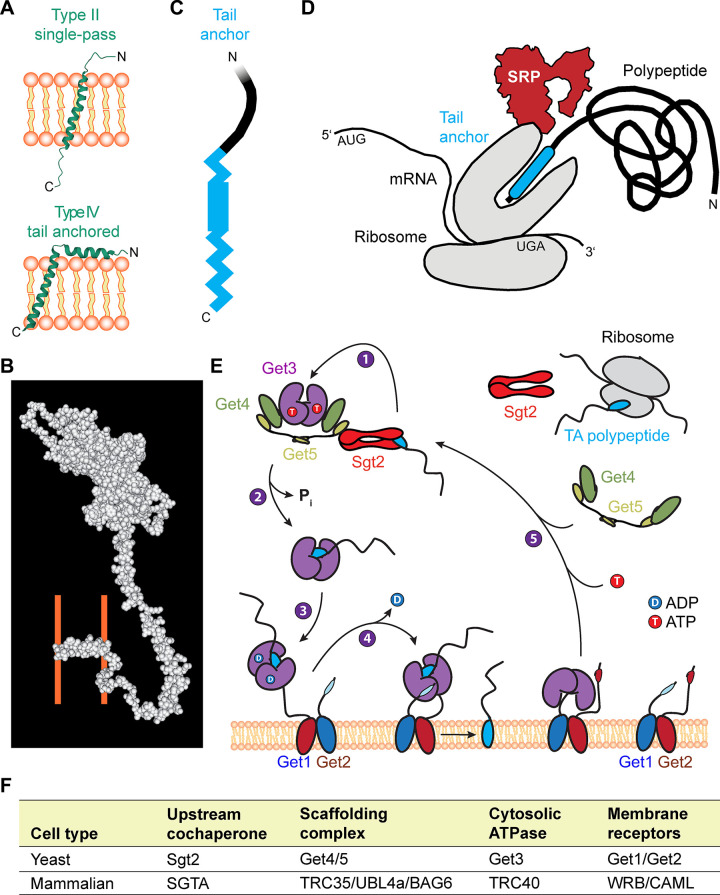
Membrane topology, biogenesis, and endo/sarcoplasmic reticulum (ER/SR) membrane insertion of tail-anchored (TA) proteins via the guided entry of TA proteins (GET) pathway. *A*: topology comparison of the single-pass type II transmembrane protein integrin-α1 (PDB 2L8S) versus the type IV TA protein phospholamban (PDB 2HYN). *B*: space filling JPH2 3-dimensional (3-D) structure atomic prediction. Wild-type junctophilin 2 (JPH2) template-based tertiary structure modeling by RaptorX and Molekel. The ER/SR membrane leaflets’ approximate positions are indicated behind the TA COOH-terminal (C) domain (orange lines). N, NH_2_ terminal. Modified JPH2 model based on the same protein sequence from Gross et al. (60) using Molekel software. Molekel is an open-source 3-dimensional molecular visualization package for analyzing the results of computational chemistry packages (61). *C*: the transmembrane domain provides the moderately to strongly hydrophobic physicochemical properties, targeting TA proteins to the ER/SR organelle (62). *D*: at the end of ribosomal biogenesis the nascent polypeptide hydrophobic COOH-terminal TA (cyan) faces the challenging aqueous cytosolic environment. SRP, signal recognition particle. *E*: GET pathway and its components established in yeast: *1*) Sgt2 loaded with the TA polypeptide docks to the closed form of the ATP-bound (T) pretargeting complex Get3-Get4-Get5; *2*) ATP hydrolysis by Get3; *3*) cytosolic translocation of the cargo-loaded Get3-TA complex to the ER membrane, and capture by the Get1-Get2 complex; *4*) Get1 interactions driving the transition of Get3 to an open conformation, concomitant ADP (D) release, insertion and release of the TA polypeptide in the ER bilayer; and *5*) ATP (T) followed by Get4-Get5 binding driving the dissociation of Get3 from the ER receptor toward its recruitment in the next cycle of TA cargo engagement [modified with permission from Borgese et al. (63)]. *F*: table summarizing the yeast and mammalian GET pathway components.

Tail-anchored (TA) proteins such as JPH1–4 form a subclass of type-II oriented integral membrane proteins that contain a single transmembrane domain at the extreme COOH terminus (FIGURE 8), whereas the NH_2_-terminal portion is oriented toward the cytoplasm (FIGURE 9*A*). Interestingly, the predicted secondary and tertiary JPH2 structures have a relatively high probability (55.7%) for a cytosolic α-helical structural fold, with a tandem of two α-helices interrupted by a short joining loop, while confirming the COOH-terminal α-helical tail (FIGURE 8) (60). A general example of a three-dimensional (3-D) space filling model visualizes the JPH2 transmembrane and cytosolic domains and their spatial localization (FIGURE 9*B*) (60). Hence, in contrast to type II single pass proteins, JPH1–4 and phospholamban (FIGURE 9, *B* and *C*) are defined as type IV proteins by their single hydrophobic membrane anchor containing the organelle-targeting information (63). Since the COOH-terminal tail of TA proteins emerges from the ribosomal tunnel only after termination of the polypeptide chain (FIGURE 9*D*), they cannot be inserted in the ER bilayer cotranslationally via the Sec61 translocon. Hence, it is very plausible that the JPH1–4 precursor protein’s TA domain is handed over by an alternative chaperone complex to a posttranslational pathway while emerging from the ribosomal tunnel, targeting it for insertion into the ER membrane (FIGURE 9*D*) (64).

The helical characteristics of the TM domain of JPH1–4 play a dominant, and for TA proteins usually exclusive role in engaging the posttranslational ER-targeting pathway (FIGURES 8 and 9*B*). Only TA proteins with a moderately to strongly hydrophobic TM tail are targeted to the ER (62). Hence, the specific helical propensity of the TM tail has an important molecular function in engaging the necessary ER-targeting factors (62, 65). Together, the hydrophobic and helical propensities of JPH1–4 thus provide the essential topogenic signal (FIGURES 8 and 9*C*), which dictates the ER destination of the TA polypeptide. However, to prevent the misfolding of the COOH-terminal tail in the challenging aqueous cytosolic environment, the signal recognition particle initially provides the posttranslational chaperone function at the ribosome for the nascent TA polypeptide, when the transmembrane domain ermerges form the exit tunnel concurrent with translation termination (FIGURE 9*D*) (59). A major route for ER insertion of TA proteins is the evolutionarily conserved guided entry of TA proteins (GET) pathway in yeast and the homologous transmembrane recognition complex (TRC) pathway in mammals (59). Therefore, while the precise ER-targeting steps remain to be established for JPH1–4, the posttranslational GET/TRC pathway has emerged as a plausible candidate.

#### 3.1.2. ER membrane insertion.

For JPH1–4 proteins, it is still unknown whether the posttranslational GET/TRC pathway provides the essential transcytosolic and ER insertion-targeting steps (59, 63, 65). The GET pathway has been thoroughly characterized in the yeast model system, providing a solid understanding of the specific substrate handover steps (for recent reviews, please see Refs. 63, 65). The recruitment of the TA substrate depends centrally on ATP hydrolysis-driven substrate cycling (FIGURE 9*E*) (63). In short, first the cochaperone Sqt2 binds and translocates the TA substrate emerging from the ribosome tunnel, forming the pretargeting complex with Get5 (FIGURE 9*E*) (63). Next, the TA substrate is handed over to the central ATPase Get3, followed by ATP hydrolysis and P_i_ release driving the translocation of the dimeric TA•Get3 complex to the ER membrane (FIGURE 9*E*) (63). Third, TA•Get3 complex interacts with the ER receptor Get1/2 complex on the ER membrane (FIGURE 9*E*). Fourth, the Get3•Get1/3 inserts the TA transmembrane domain into the bilayer, while ADP is released (FIGURE 9*E*). Fifth, upon ATP binding the Get3 dimer is released, completing its cycling through assembly of the next pretargeting complex (FIGURE 9*E*) (63). Based on robust understanding of the GET pathway, yeast may represent one suitable major model system to investigate homologous JPH1- to JPH4-targeting mechanisms.

The mammalian TRC pathway homologous components more recently identified are compared with yeast in FIGURE 9*F* (63). In analogy the nascent COOH-terminal tail is captured by the ribosome-associated chaperone pretargeting complex (TRC35/UBL4A/BAG6) for handover to the ATPase TRC40. Following P_i_ release, the TA•SGTA dimer provides the ER-targeting route for the TA precursor in higher eukaryotes. TRC40 further hands the TA polypeptide over to the ER receptor complex formed by WRB (tryptophan-rich basic protein) (66) and CAML (calcium signal-modulating cyclophilin ligand) (67). Following capture and handover by TRC40, the receptor complex WRB/CAML inserts the TA in the ER bilayer while ADP is concomitantly released (68). Finally, ATP binding and cytosolic interactions with URC35/UBL4A drive the dissociation of TRC40 from the ER receptor, restoring the TA-targeting cycle (64). Recently, tissue-specific WRB knockout mice have revealed that the targeting of the mammalian TA protein syntaxin-5 in cardiomyocytes and hepatocytes not only depends on the TRC40 pathway but cannot be sufficiently predicted in vitro in yeast (64). While for JPH1–4 targeting by the TRC40 pathway has been neither refuted nor established, the former study identifies important limitations of the yeast model, emphasizing the importance of rodent systems to fully explore the mammalian ER/SR-targeting pathway and its role in genetic diseases and in vitro studies, where mutations in TA proteins can lead to ER escape potentially compromising other organelles (59).

### 3.2. Membrane Tethering and Subcellular Clustering

#### 3.2.1. Mechanisms of junctophilin membrane tethering.

The conserved eight NH_2_-terminal MORN domains (FIGURES 1 and 8) were proposed to provide the surface membrane-binding capacity of JPH1–4 (2, 13, 27, 69). For binding to the negatively charged phospholipid head groups in the cytosolic plasma membrane (PM) leaflet, the NH_2_-terminal MORN domain cluster 1–6, as well as the more COOH-terminal cluster 7–8, provide numerous positively charged residues. Vice versa, to function as a solute barrier, the mammalian PM forms a thick bilayer with tightly packed lipids providing negative cytosolic surface charges. Sphingolipids and sterols are particularly abundant in the PM, making the mammalian bilayer a particularly thick and rigid barrier (70). While a protein-lipid binding assay with a purified human JPH2 lacking the transmembrane domain (JPH2-ΔTM) did not bind cholesterol, it revealed the binding of abundant phospholipids, such as phosphatidylserine (71). Other phospholipids that bind to full-length human JPH2 or JPH2-ΔTM include phosphoinositides, such as phosphatidylinositol-3-phosphate [PtdIns(3–5)*P*_3_] (71). Whereas phosphatidylserine is physiologically located in the cytoplasmic PM leaflet, interestingly it migrates to the outer PM leaflet in apoptotic or stressed cells, for example, ischemic cardiomyocytes (72). Moreover, it has been suggested that this phosphatidylserine migration to the outer PM leaflet disrupts the binding of JPH2 to the junctional transverse (T)-tubule membrane (72). Together, these studies agree with the pioneering work by Takeshima et al. (2) identifying the MORN motifs as a potential JPH1 binding mechanism to the PM of skeletal muscle fibers.

Synaptotagmin-3 (Syt3) functions as Ca^2+^ sensor in Ca^2+^-dependent exocytosis of secretory vesicles, inducing its binding both to phospholipid membranes and assembled SNARE complexes. E-Syt3, a ubiquitously expressed synaptotagmin, promotes the formation of cortical ER contacts in eukaryotic cells through binding to the phosphoinositide PtdIns(4,5)*P*_2_ via its COOH-terminal C2 domain (69). When overexpressed in adult mouse flexor digitorum brevis (FDB) muscle fibers, green fluorescent protein (GFP)-JPH1 and GFP-JPH2 selectively localize to this cell type-specific triadic junctional membrane contacts (69). Interestingly, in mature multinuclear FDB muscle fibers, the TM domain-deleted GFP-JPH1-ΔTM fusion protein showed a preserved PM binding and colocalization with PtdIns(4,5)*P*_2_, indicating similar requirements as compared with E-Syt3 (69). These observations established that the MORN domains may mediate the sarcolemmal PM binding at T-tubule PM invaginations together with PtdIns(4,5)*P*_2_ in mature skeletal muscle fibers (69).

Interestingly, partial or complete MORN domain deletion in GFP-JPH1ΔMORN1–6- or GFP-JPH1ΔMORN1–8-deleted constructs revealed that the fluorescent proteins remain localized at triadic junctions in cultured FDB muscle fibers, possibly due to homo- or heterodimerization with JPH1 or JPH2, respectively (73). As JPH1 and JPH2 are concentrated locally in subcellular clusters in cardiomyocytes and skeletal myofibers (73–75), weak protein/lipid, lipid/lipid, and protein/protein interactions may jointly stabilize the cell type-specific local nanodomain composition and PM binding (70). Hence, JPH1 clustering and tethering to contact sites at the inner PM leaflet segregate specific phospholipids in local rafts (76). Such phospholipid rafts might be functionally important for the molecular association of JPH1 with certain ion channels to stabilize and control their local function, for example, the voltage-gated L-type Ca_V_1.1 and Ca_V_1.2 channels in skeletal and cardiac muscle cells further discussed below (47). Thus, while it appears that MORN domains are important for JPH binding to the PM, JPH proteins lacking MORN domains may still localize to the correct nanodomain due to homo- or heterodimerization or binding to other proteins within the JMC (73).

#### 3.2.2. Structural features of MORN domains.

MORN domains are defined as possible plasma membrane-binding motifs in junctophilins and PIP5K protein kinases according to the SMART protein database (53). The existence of MORN domains within human proteins is of considerable interest, since β-hairpin-based MORN tandem repeats function either as versatile lipid-binding or protein-protein interaction modules, compared with other classes of ubiquitous tandem repeats occurring in over 14% of all proteins (77); however, relatively little is known about the structurally defining features, since only a few MORN β-sheet repeat-containing proteins have been characterized in depth.

Bioinformatic sequence analyses revealed that each of the four JPH1–4 isoforms contains 14-aa long repeats with YEGEWxNGKxHGYG as a consensus motif (27). Indeed, an extended sequence comparison of the MORN-I domain of JPH2 throughout species showed a high degree of conservation (FIGURE 6). A recent genome-wide bioinformatic analysis concluded that MORN repeat proteins are ubiquitously expressed throughout eukaryotes and prokaryotes (78) (FIGURE 7). In contrast to the MORN domains found in JPH1–4, a principally different 23-aa long repeat architecture based on a highly conserved GxG12-14 motif has been revealed as the consensus motif for MORN proteins of certain plants and parasites (28, 79).

Functionally, the MORN domain found in JPH isoforms mediate interactions with lipids. A recent in vitro lipid-binding study of over 100 proteins of the protozoan ciliate *Tetrahymena thermophila* confirmed the lipid-binding capacity for similar 14-aa long MORN consensus motifs (80). In contrast, it appears that the negative surface charges on the parasite MORN domains exclude a phospholipid-binding function, both rather mediate binding to specific cytoskeletal protein domains (81). However, both parasitic and mammalian MORN domain structures are capable of binding specific cytoskeletal protein domains as discussed in sect. 2.2.3 (81).

**FIGURE 10.**
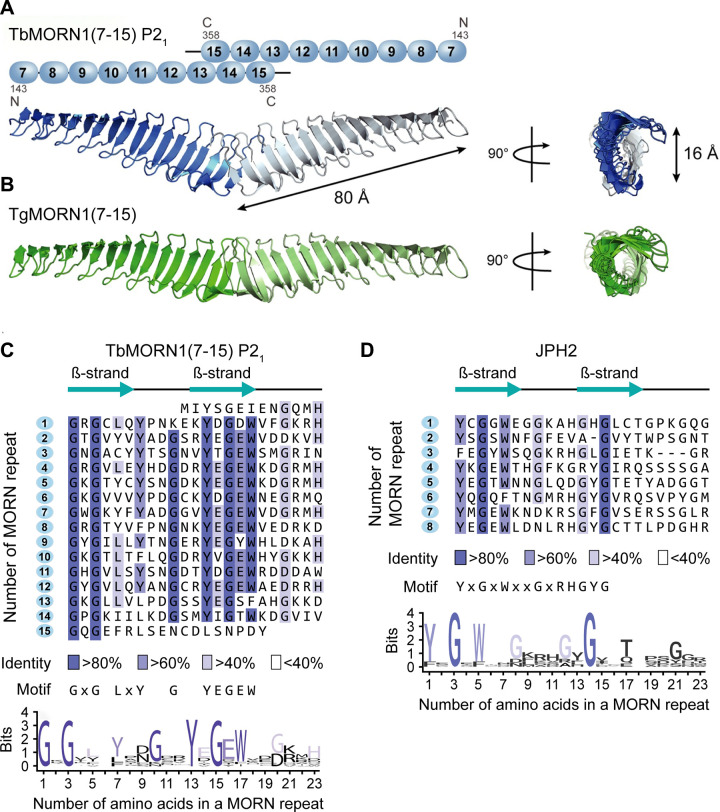
Conserved 23-amino acid tandem membrane occupation and recognition nexus (MORN) repeat atomic structures and predicted junctophilin 2 (JPH2) β-strand architecture. *A* and *B*: Domain depiction and crystal structure of the NH_2_-terminal deletion proteins *Trypanosoma brucei* TbMORN1(7–15) and *Toxoplasma gondii* TgMORN1(7-15) each forming tail-to-tail homodimers. Amino acid numbers and NH_2_(N)/COOH (C) termini are indicated on *top*. The crystal structure is shown both from the side and 90° rotated as indicated. Major dimensions are indicated (double arrows). Each truncated protomer contains 9 MORN repeats of which the 3 COOH-terminal repeats additionally provide the antiparallel tail-to-tail interactions. The secondary structure consists exclusively of antiparallel β-strands and peripheral loops. TbMORN1(7–15) and TgMORN1(7–15) exhibit the same number of MORN repeats and structural configurations. Modified with permission from Sajko et al. (81). *C*: consensus MORN repeat sequence of TbMORN(7–15) revised according to its crystal structure. While repeats 7–15 exist in the crystal structure, the deleted repeats 1–6 are inferred. Blue color intensities indicate the conservation of sequence identity as indicated by the legend (%cutoff). TbMORN1 consists entirely of MORN repeats and β-hairpins, where the NH_2_-terminal and COOH-terminal 6-residue β-strands are connected by a 5-residue loop. Finally, a 6-residue loop connects to the subsequent MORN repeat. The highly conserved GxG and additional motifs of the 23-residues consensus MORN repeat are indicated below. Modified with permission from Sajko et al. (81). *D*: conserved JPH2 YxGxW and GxG motifs of the 23-residues consensus MORN repeat sequence of JPH2. While the JPH2 sequence identity indicated by %cutoff (legend) is lower, the 6-residue tandem β-strands connected by a 5-residue loop are confirmed by similarity to the revised TbMORN1 consensus sequence.

The specific structures of the MORN domains found in JPH isoforms have not been elucidated. On the other hand, the MORN structure of TbMORN1(7–15) from the eukaryotic blood parasite *Trypanosoma brucei* was solved. This cytoskeleton-binding protein complex consists of 15 repeats each forming a 17-aa long MORN motif regularly connected by 6-aa short loops (FIGURE 10*A*) (81). Moreover, both the crystal structures of an NH_2_-terminal deletion construct containing 9 MORN repeats TbMORN1(7–15) and its *Toxoplasma gondii* homologue TgMORN1(7–15) demonstrate highly conserved elongated twisted β-hairpin sheets, which interact tail-to-tail in antiparallel dimers through a COOH-terminal multi-MORN domain-dependent mechanism (FIGURE 10, *A* and *B*) (81).

The sequence alignment of the 15 MORN repeats was analyzed taken in consideration structural data, and highly conserved GxG and YEGEW motifs in the first and second β-strand, respectively (FIGURE 10*C*) (81). When we aligned the 8 MORN domains in JPH2 in a similar way, we identified two β-strands within and extending the conserved YxGxWxxGxRHGYG motif (FIGURE 10*D*). Whereas the mammalian and paraside MORN domains appear to be divergent in terms of their consensus motifs and lipid-binding abilities, it may be possible that there are protein folding similarities based on the presence of a dual β-strand organization in both types of MORN domains. However, future structural studies are needed to uncover the precise protein folding and membrane binding properties of the MORN repeats in JPH1–4.

#### 3.2.3. Subcellular junctophilin clustering.

**FIGURE 11.**
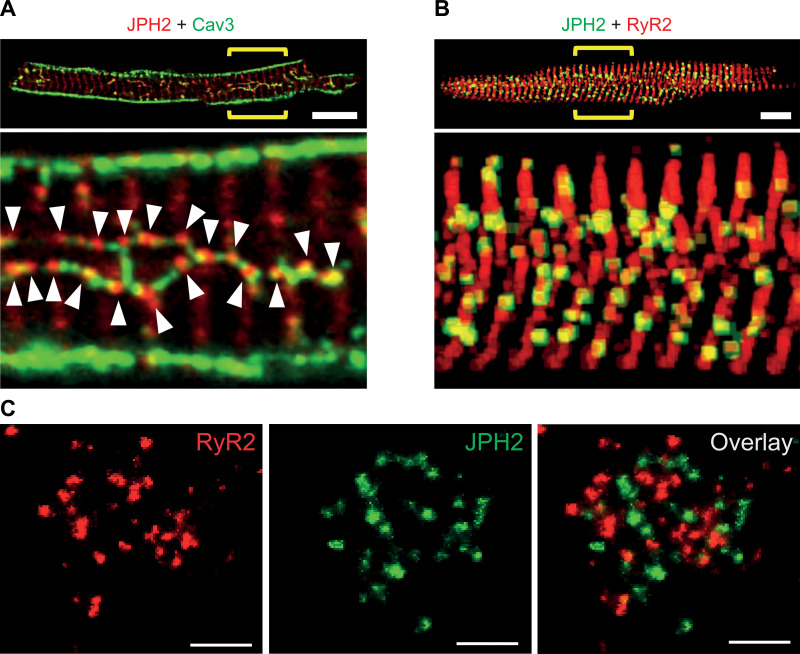
Subcellular junctophilin 2 (JPH2) clustering in atrial and ventricular cardiomyocytes. *A*: mouse atrial cardiomyocyte confocal section overview (*top*) and magnified image region indicated by yellow brackets (*bottom*). Magnification showing the variable subcellular signal qualities of immunostained JPH2 clusters. Larger JPH2 cluster signals (red) marked by white arrowheads are mainly located at deep intracellular axial tubule structures labeled with caveolin-3 (green). Less intense JPH2 signals apparently localize to transverse striations devoid of Cav3 signals. Scale bar = 10 μm. Modified with permission from Brandenburg et al. (82). *B*: confocal 3-dimensional z-stack projection overview (*top*) and magnification (*bottom*) showing intense JPH2 cluster signals (green) mainly deep inside a mouse atrial cardiomyocyte, occasionally intersecting ryanodine receptor 2 (RyR2) channel clusters (red) in transversal striations evident by colocalized signals (yellow). Scale bar = 10 μm. Modified with permission from Brandenburg et al. (82). *C*: high-power magnification of rat ventricular myocyte. Exchange-PAINT quantitative superresolution imaging of a single cluster of RyR2 channels (red), interspersed JPH2 signals (green), and signal overlay (*right*). Scale bars = 200 nm. Modified with permission from Jayasinghe et al. (75).

Junctophilins bind not only to other proteins and lipids but are also known for their self-binding capacity. Homologous self-interactions may thus provide the molecular mechanism for subcellular clustering within JMCs. The features and mechanisms of junctophilin clustering will be discussed in this section. Junctophilins are anchored into the ER/SR membrane using conserved COOH-terminal transmembrane tail-anchors (FIGURES 8 and 9) (13, 27). As the α-helical region contains on average 70 aa, it bridges the discontinuous membrane contact subspace in the dyadic or triadic junction in cardiac and skeletal muscle cells, respectively. Single-molecule transmission electron microscopy imaging of truncated soluble and full-length human JPH2 showed filament-like elongated structures of ∼15 nm in length (71). Hence JPH1 and JPH2 can physically bridge the triadic or dyadic gap in skeletal myofibers or cardiomyocytes, respectively. Whereas JPH2 is highly colocalized with RyR2 channels in clusters throughout the SR network of adult ventricular myocytes (74), atrial cardiomyocytes express ∼70% less JPH2 protein in the mouse heart (82). Nonetheless, large JPH2 clusters exist in atrial cardiomyocytes exclusively in the junctional dyads of endomembrane Transverse-axial-tubule network structures, such as the predominant axial tubules (FIGURE 11*A*) (82). In contrast, ∼75% of the non-junctional atrial RyR2 clusters devoid of any PM contact are associated with weak or no JPH2 immunofluorescence signals. 3-D reconstruction confirmed the non-random distribution of JPH2 clusters and the curvilinear RyR2 coclustering consistent with a primary localization at axial tubules in atrial cardiomyocytes (FIGURE 11*B*) (82).

In contrast, true nanoscale superresolution imaging demonstrated the local molecular JPH2 stoichiometry relative to RyR2 channels within the same junctional nanodomain in rat ventricular cardiomyocytes, confirming the typical distribution of the highly coclustered proteins in situ (FIGURE 11*C*) (75, 83). However, unstimulated atrial cardiomyocytes depend functionally on SR Ca^2+^ release solely via the junctional RyR2 channels coclustered with JPH2 to activate contraction through in a more rapid manner also known as atrial “kick” during the last phase of ventricular filling (84). While the junctional RyR2 clusters are highly phosphorylated by PKA and CaMKII in situ under unstimulated baseline conditions exclusively in atrial cardiomyocytes, a much greater fraction of non-junctional RyR2 clusters become PKA phosphorylated only following β-adrenergic stimulation (82, 84). Together, these studies revealed major differences in the proteomic composition, cell biology, and physiology of atrial compared with ventricular cardiomyocytes based at least in part on the differential expression of JPH2.

**FIGURE 12.**
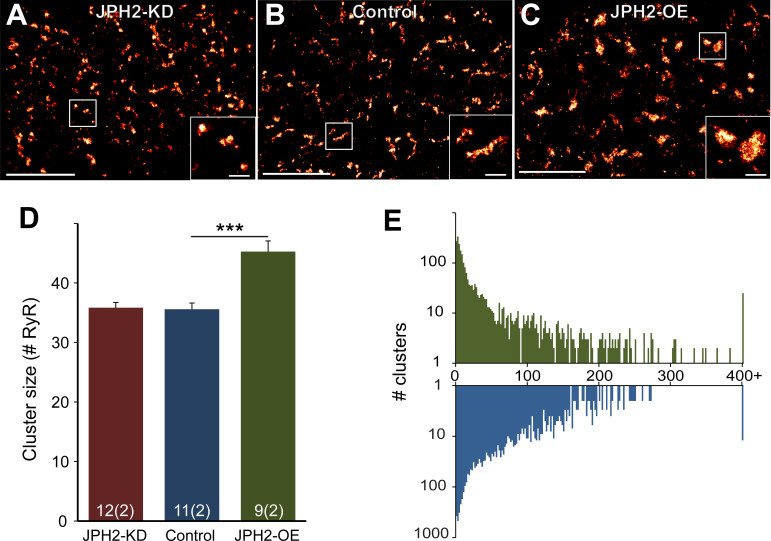
Subcellular ryanodine receptor 2 (RyR2) clustering in ventricular control and transgenic heart sections from adult mice. *A-C*: superresolution images showing immunolabeled RyR2 channel clusters in left-ventricular myocardial sections at transverse striations. *A*: junctophilin 2 (JPH2) knockdown (JPH2-KD). *B*: control wild-type heart section. *C*: JPH2 overexpression (JPH2-OE). Magnified views of singular clusters are shown in the *insets*. Scale bars = 4 µm in main panels; scale bars = 0.5 µm in *insets* indicated by white rectangles. *D*: RyR2 cluster size. *E*: number of RyR2 clusters. Mouse strains are indicated by color. Control, *n* = 11 cells, 2 animals; JPH2-KD, *n* = 12 cells, 2 animals; JPH2-OE, *n* = 9 cells, 2 animals. Data are displayed as means ± SE. ***P < 0.001 (Kruskal-Wallis two-sided test). Modified with permission from Munro et al. (74).

Germline knockout of JPH2 not only disrupts the fixed junctional intermembrane gap spacing in the murine cardiomyocytes, but is embryonically lethal, identifying JPH2 as an essential cardiac gene (2). Mathematical 3-D superresolution modeling of Ca^2+^ sparks based on a realistic molecular and spatial junctional composition demonstrated quantitatively how an increased spacing of the junctional gap width beyond 15 nm profoundly diminishes the fidelity of spontaneous Ca^2+^ spark firing underlying excitation-contraction coupling (85). Cardiomyocyte-restricted tamoxifen-inducible short hairpin RNA (shRNA)-mediated JPH2 knockdown resulted in rapid-onset severe heart failure and increased mortality within 1 wk in adult mice. A diminished junctional JPH2 clustering, an increased junctional dyad spacing, and RyR2 channel dysfunction leading to increased SR Ca^2+^ leak occurred as a result of disrupted JMCs in cardiomyocytes (6). Interestingly, side-by-side comparison of immunolabeled clusters of RyR2 channels in ventricular rat cardiomyocytes by superresolution imaging in JPH2 knockdown versus JPH2 overexpression mice showed quantitative changes only for the latter, specifically an increased RyR2 cluster size (FIGURE 12) (74). In summary, whereas JPH2 is highly coclustered with RyR2 channels in ventricular cardiomyocytes in JMCs at transverse tubules, JPH2 knockdown leads to an irregular and increased junctional gap spacing with increased SR Ca^2+^ leak.

#### 3.2.4. Homomeric and heteromeric junctophilin self-interactions.

Emerging evidence suggests that JPH1 can self-interact with JPH1 or JPH2 isoforms (69). Overexpression of the GFP fusion protein GFP-TM-JPH1, containing only the COOH-terminal 26-aa tail of JPH1, in adult FDB skeletal muscle fibers in culture resulted in typical triadic junction localization of the fluorescent protein (69). This finding indicates that the TM domain contains molecular determinants for local JPH1 clustering in organotypic triadic junctions of the SR organelle at sarcomeric Z-disks. Bimolecular fluorescence complementation studies revealed a fluorescent signal in primary rat myofibers overexpressing two complementary Venus-tagged JPH1-TM fusion proteins, indicative of dimerization (69). Moreover, performing the same fluorescence complementation assay in HeLa cells reconstituted the dimerization of JPH1 in the ER outside the muscle-specific environment (69). Fluorescence recovery after photobleaching (FRAP) imaging of overexpressed GFP-JPH1 revealed a reduced mobility at triad junctions in mature differentiated multinuclear muscle fibers (86). In differentiated myofibers expressing a low level of GFP fluorescence, the mobile fraction of full-length GFP-JPH1 was relatively low (32.3 ± 12.6%), whereas JPH1 lacking all eight MORN domains or the TM domain were significantly increased (69). Interestingly, FRAP imaging in non-muscle cells showed that the mobile fraction of full-length GFP-JPH1 was significantly higher compared with differentiated muscle fibers (86) Together, these findings suggests that the lower dynamic mobility of the GFP-JPH1 full-length fusion protein at the skeletal muscle-specific triad contact sites occurs both through self-interactions and bilayer interactions within JMCs.

Coimmunoprecipitation followed by immunoblotting further showed that JPH1 and JPH2 can form both homodimers and heterodimers (69). HEK293 cell transfection of Myc- or GFP-tagged JPH1 and JPH2 for coimmunoprecipitation analysis demonstrated that both JPH1 and JPH2 interact each in homodimers but also heterodimers (69). Additionally, GST-tagged fusion proteins containing the joining region of JPH1 or JPH2 pulled-down the native mouse JPH1 and JPH2 microsomal proteins from skeletal muscles lysates (69). Hence, the JPH1 or JPH2 joining region appears to stabilize both the homomeric and the heteromeric dimer formation. Together with complementary studies in HEK293 cells, this established that both homomeric and heteromeric JPH1 and JPH2 interactions can occur.

### 3.3. Junctophilin Interactions and Subcellular Functions

#### 3.3.1. Ca_V_1.1/junctophilin interactions in skeletal muscle.

**FIGURE 13.**
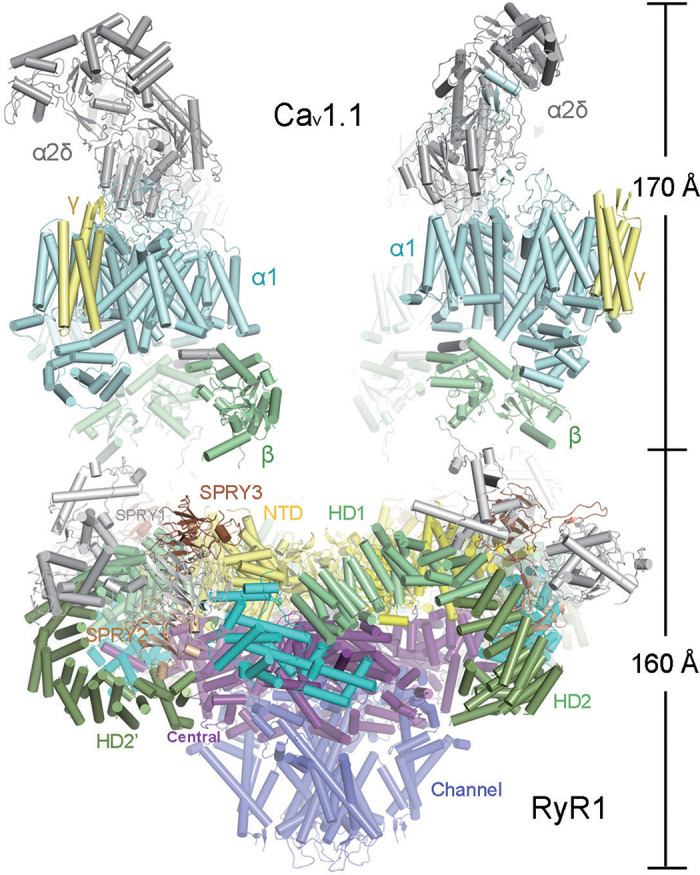
Molecular mechanisms of skeletal MCS excitation-contraction coupling. Side view showing a speculative model of the macromolecular complex containing 2 Ca_V_1.1-α1S channels (PDB 3JBR) and 1 ryanodine receptor 2 (RyR2) channel (PDB 3J8H). Multiple SPRY1/3 domains on the cytoplasmic portion of the RyR1 tetramer may mediate the physical coupling with Ca_V_1.1 as indicated [NH_2_-terminal domain (NTD)]. Vice versa, the voltage-dependent opening of the Cav1.1-α1S pore may induce conformational changes of the β-subunit and the II-III linker, and the latter may trigger conformational interactions through the SPRY1/3 domains of the RyR1 tetramer. Modified with permission from Bai et al. (87).

In mature skeletal myofibers, the voltage-gated Ca_V_1.1 LTCC activates excitation-contraction (E-C) coupling in skeletal muscles through direct physical interactions with the ryanodine receptor type 1 (RyR1) (FIGURE 13). These two types of Ca^2+^ channels are kept at a ∼12-nm distance by JPH1 and JPH2, that stabilize the skeletal myofiber JMCs through multiple mechanisms: *1*) tethering of the cytosolic PM leaflet at sarcolemmal transverse tubule invaginations to the junctional SR membrane, and *2*) through interactions with the pore-forming Ca_V_1.1-α1S and its ancillary β1/α2δ/γ-subunits (47).

**FIGURE 14.**
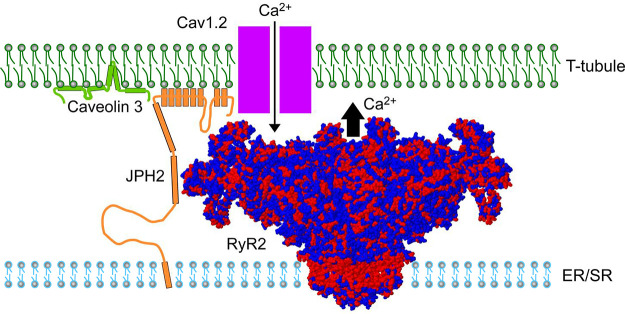
Cardiac muscle MCS excitation-contraction coupling. Cartoon showing a side view of the molecular junctional membrane complex components. Structured junctophilin 2 (JPH2) domains are indicated by orange boxes, according to Gross et al. (60). Caveolin 3 model following Parton et al. (88). Space-filling structure of the ryanodine receptor 2 (RyR2) channel open state and its functional relation to both the Cav1.2 channel in the T-tubule and the endo/sarcoplasmic reticulum (ER/SR) transmembrane domain (PDB 5GOA) [modified from Peng et al. (89)]. Arrows indicate Ca^2+^-induced Ca^2+^ release. RyR2 surface colors: blue, hydrophilic; red, hydrophobic.

Upon membrane depolarization, Ca_V_1.1-α1S channel opening is transmitted to its cytosolic structures in skeletal muscle fibers independent of extracellular Ca^2+^ influx. It has been speculated that the conformational changes of the cytosolic domains of a pair of Ca_V_1.1-α1S channels, their β-subunits and perhaps the cytosolic II–III loops, may directly trigger the opening of the RyR1 channel tetramer through depolarization-triggered conformation-dependent interactions (FIGURE 14) (90). Indeed, in mature skeletal myofibers, multichannel assemblies of Ca_V_1.1 and RyR1 cluster locally in triad junction MCS, where the intracellular transverse tubule membrane is closely juxtapositioned to the junctional SR cisternae (91). Collectively, one triadic MCS assembly composed of the macromolecular Ca_V_1.1 and RyR1 channel clusters and their adjoining PM and SR lipid rafts hence define the molecular JMC composition in the different subtypes of skeletal muscles. For correct targeting of Ca_V_1.1 channels to the JMC in cultured skeletal muscle myoblasts, a triad-targeting signal in the 55-aa sequence 1607–1661 was sufficient for targeting and clustering of the neuronal Ca_V_1.1-α1A isoform into junctional triads (92). Furthermore, using immunoprecipitation and pull-down assays, Golini et al. (93) identified amino acids 230–369 in JPH1 and 216–399 in JPH2, respectively, as sufficient determinant for the association with the Cav1.1-α1S pore subunit.

JPH1 and JPH2 are both expressed in skeletal muscle albeit the former more abundantly (2). Overexpression of a TM domain truncated JPH1ΔTM protein was sufficient for binding to the PM both in *Xenopus oocytes* and canine kidney cells (2). On the other hand, siRNA-mediated knockdown of JPH1 or JPH2 in immature cultured myotubes disrupted Ca_V_1.1 channel clustering in peripheral JMCs at the cell surface and diminished electrically evoked Ca^2+^ transients, while the junctional membrane structure remains intact (47). Coimmunoprecipitation and GST pull-down demonstrated a physical interaction of JPH1 and JPH2 through a conserved 12-aa motif in the proximal cytosolic COOH-terminal domain of the skeletal muscle Ca_V_1.1-α1S and the cardiac Ca_V_1.2-α1C pore subunits (47). In differentiated skeletal myotubes transfected with a TM-deleted and FLAG-tagged plasmid, the JPH1ΔTM-FLAG fusion protein binds to the sarcolemmal T-tubule but no longer to the SR membrane (47). Together, these studies established that JPH1 and JPH2 recruit the Ca_V_1.1-α1S pore subunit through a COOH-terminal binding motif to JMCs and tether the cytosolic PM leaflet stabilizing the JMC structure and function locally in the Ca^2+^ release nanodomain.

#### 3.3.2. Cardiac Ca_V_1.2 and Ca_V_1.3 interactions with junctophilin.

JPH2 is the major cardiac isoform that stabilizes the cardiomyocyte dyadic nanodomains in a cell type-specific fashion maintaining the intermembrane subspace distance of 12–15 nm between the transverse tubule and the junctional SR membrane (2, 84). In contrast to skeletal muscle cells, in ventricular cardiomyocytes the Ca_V_1.2-α1C channel pore in T-tubules and the ryanodine receptor type 2 (RyR2) are located in nanometric proximity, such that E-C coupling in the dyadic subspace occurs indirectly through Ca^2+^-induced Ca^2+^ release (CICR) (FIGURE 14). For local CICR control, both the atrial and ventricular dyadic structures are stabilized by the membrane tethering, RyR2 coclustering, and scaffolding functions of JPH2 (2, 6, 82). Thus local JPH2 coclustering with RyR2 and binding to Ca_V_1.2-α1C define the proteomic JMC nanodomain constitution, which underpins the efficacy of cardiac E-C coupling during each heartbeat (94, 95). Importantly, JPH2 directly modulates the activity of the RyR2 channel by stabilizing the channel closed state in the resting cell during diastolic relaxation (6, 75, 82, 96).

As discussed above, the JPH2 coclustering with RyR2 stabilizes the junctional SR function locally and the relative slow local Ca^2+^ signaling during CICR and cardiac E-C coupling both in atrial and ventricular cardiomyocytes (FIGURE 11, *A* and *C*). Recently, the molecular mechanism responsible for Ca_V_1.2 channel recruitment to the cardiac SR Ca^2+^ release unit has been identified. The cytosolic JPH2 joining region between the NH_2_-terminal and COOH-terminal MORN domain clusters was shown to be in nanometric proximity to the Ca_V_1.2-α1C pore subunit in isolated adult feline ventricular cardiomyocytes (60). Moreover, coimmunoprecipitation followed by immunoblotting demonstrated a molecular interaction between the cardiac JPH2 and the LTCC-α1C proteins (60). Interestingly, inducible overexpression of a mutant JPH2 proteoform with seven random point mutations introducing charged polar amino acids in the joining region (7mutPG1-JPH2) in cultured feline ventricular cardiomyocytes decreased the Ca_V_1.2-α1C interaction with the native JPH2 protein by ∼30–40%, resulting in a decrease both of the dyad frequency and the density of the transverse tubule components (60). Moreover, while 7mutPG1-JPH2 overexpression did not change the Ca^2+^ transient at baseline, it caused proarrhythmic Ca^2+^ waves following β-adrenergic stimulation of cultured ventricular cardiomyocytes.

Coimmunoprecipitation of JPH2 confirmed several ion channels as interactors including Ca_V_1.2-α1C in ventricular heart tissue (60, 97). Recently, the JPH2 interaction analysis was extended to the atrial heart tissue of genetically modified mouse models (82, 98). Whereas shRNA-mediated JPH2 knockdown disrupts the RyR2 coclustering in mouse atrial cardiomyocytes, transgenic JPH2 overexpression increases the junctional RyR2 cluster size and even induces the biogenesis of large 3-D poly-adic JMCs (82). In addition, atrial cardiomyocytes are known to express an additional LTCC isoform, Ca_V_1.3-α1C, with distinct biophysical voltage-gating properties (99). Recently, the cell-type specific Ca_V_1.3 channel clustering in and near the axial tubule endomembrane network was demonstrated by superresolution immunofluorescence imaging in atrial cardiomyocytes (84). Hence, future studies will need to elucidate the precise subcellular roles of the differential atrial JPH2 interactions with both the Ca_V_1.2-α1C and Ca_V_1.3-α1C isoforms.

#### 3.3.3. Ca_V_1.2/junctophilin interactions in smooth muscle.

The SR and PM form stable peripheral MCS sites in contraction competent vascular smooth muscle cells (100, 101). These subcellular contact sites support the local Ca^2+^ signals that are functionally important for the regulation of the membrane potential and the contractile behavior of smooth muscle cells (101). In the surface JMCs, RyR2 channel clusters are functionally coupled with large-conductance Ca^2+^-sensitive K^+^ (BK) channels such that a single local Ca^2+^ spark signal induces a large transient outward K^+^ current, hyperpolarizing the plasma membrane and deactivating the voltage-dependent Ca^2+^ influx to induce arterial relaxation (102–104). This local Ca^2+^ signaling mechanism provides a negative feedback regulation that limits the magnitude and duration of cerebral artery constriction (104, 105). A recent study identified JPH2 as the most abundant isoform in native smooth muscle cells isolated from cerebral arteries (106). Acute JPH2 knockdown diminishes the site volume of the MCS between the SR and plasma membrane in arterial smooth muscle cells. Morpholino treatment of arterial smooth muscle cells and patch clamping demonstrated that JPH2 knockdown leads to a loss of Ca^2+^ spark-activated BK channel activity, preventing arterial relaxation (106). In summary, reduced expression of JPH2 in arterial smooth muscle cells may increase arterial contractility and vascular resistance, potentially contributing to systemic hypertension.

#### 3.3.4. Junctophilin interactions with ryanodine receptors.

As discussed above, JPH1 directly interacts with the *RYR1*-encoded ryanodine receptor type 1 (RyR1), which mediates the rapid release of Ca^2+^ from the SR in skeletal muscles. Highly reactive thiol groups sensitive to oxidation can alter the JPH1 interaction and RyR1 function. This suggests that SR Ca^2+^ release via RyR1 is mediated by in an oxidation-dependent fashion and direct interactions with JPH1 (107). In addition, coimmunoprecipitation experiments have identified the RyR2 channel as a JPH2 binding partner (6). Junctophilin and RyR2 both are highly concentrated in clusters inside cardiac JMCs (FIGURE 11*C*) (74, 75). A disease-associated JPH2 variant, E169K, reduces the binding of JPH2 to RyR2, suggesting that residue E169 is located within the protein-protein binding domain (96).

#### 3.3.5. Neuronal junctophilin expression and interactors.

In the brain, functional cross talk between cell-surface and intracellular channels occurs in “subsurface cisterns.” JPH3 belongs to the trimeric JMC implicated in the regulation of neuronal excitability, which is involved in the formation of the junctional MCS between voltage-gated ion channels and RyR Ca^2+^ release channels. In the brain, JPH3 and JPH4 are frequently coexpressed (14). Both isoforms are most highly expressed in the caudate putamen, the granule cells of the cerebellum, the hippocampus, the nucleus accumbens, and the olfactory bulb and anterior olfactory nuclei. Additionally, JPH3 is less abundant in the ventrolateral, ventroposterior, and posterior thalamic nuclei and spinal gray matter, whereas JPH4 is undetectable in these regions (14).

Double JPH3–4 knockout mice develop a phenotype of an impaired memory and irregular hindlimb reflexes (108). Electrophysiological whole cell current-clamp recordings demonstrated that activation of the small-conductance Ca^2+^-activated K^+^ (SK) channels induced an afterhyperpolarization in hippocampal neurons requiring ER Ca^2+^ release through RyR channels. This process is physiologically triggered by *N*-methyl-d-aspartate (NMDA) receptor-mediated Ca^2+^ influx but completely absent in JPH3–4 knockout cells (108). Coimmunoprecipitation showed an interaction between JPH3 and STK23, a serine/threonine kinase that specifically phosphorylates its substrates at serine residues located in regions rich in arginine/serine dipeptides, such as RS domains (109). Vice versa, JPH3 was recognized as a specific protein phosphatase PP1α interactor, abundant in the human brain, by yeast-two-hybrid screening (110). Since the PP1 and STK23 interactions are highly context specific and mediate particular functions in cells, JPH3 might be regulated in a unique neuron-restricted fashion to control the electrical excitability of neurons in different brain tissues.

#### 3.3.6. Caveolin-3 interactions with JPH2.

Early work suggested that JPH2 might associate with the atypical, partly membrane-integral and cytosolic muscle-specific caveolin-3 protein (111). Caveolin-3 functions as a cholesterol-binding multimeric scaffolding complex, which stabilizes the omega-shaped membrane invaginations known as caveolae (112). A JPH2 interaction with the cardiac Ca_V_1.2-α1C channel and caveolin-3 was proposed based on cultured isolated adult rat cardiomyocyte immunostaining, proximity ligation, and confocal imaging. Proximity based ligation of the antibody-labeled JPH2 and caveolin-3 established a nanometric protein association in cultured rat cardiomyocytes (113). Smaller caveolin-3 scaffolds stabilize the function of multiple membrane transporters including the voltage-dependent cardiac Na_V_1.5 channel and the monocarboxylate lactate/pyruvate shuttle McT1 in ventricular cardiomyocytes (112). Immunoprecipitation experiments confirmed multiple caveolin-3-specific transmembrane protein interactions, raising the possibility that JPH2 is additionally anchored at the cytosolic PM leaflet through direct or indirect binding to caveolin-3 scaffolds. Furthermore, cholesterol-rich nanodomains the size of caveolae in living atrial cardiomyocytes and corresponding caveolin-3 immunofluorescence signal spots, as well as frequent spatial juxtapositions of caveolin-3 and JPH2 clusters were demonstrated in the axial tubule endomembrane network by superresolution microscopy. Caveolin-3 clusters can exist both in caveolae and in non-caveolar membrane domains in transverse tubule invaginations in ventricular cardiomyocytes (5, 114), where different complex forms of scaffold multimers may interact with JPH2 clusters. Interestingly, caveolae have not been observed at sites where transverse tubules and the SR membrane form JMCs in cardiomyocytes (115). In contrast, peripheral JMCs at the outer surface membrane of cardiomyocytes occur both in caveolae and flat lipid rafts (5).

#### 3.3.7. Regulation by the SPEG kinase.

Quick et al. (116) identified striated muscle preferentially expressed protein kinase (SPEG) as a novel JPH2 binding partner using mass spectrometry analysis of JPH2 immunoprecipitated from mouse hearts. To validate that SPEG directly binds to JPH2, SPEG was immunoprecipitated from mouse heart lysate and JPH2 was identified as its binding partner, confirming the results. In addition, coexpression studies of SPEG fragments and JPH2 in HEK293 cells revealed that the NH_2_-terminal domain of SPEG mediates its binding to JPH2 (110). On the other hand, it remains unknown to what part of JPH2 binding of SPEG occurs. The same study also demonstrated that SPEG can phosphorylate JPH2, at a residue that remains to be identified (116). Interestingly, the number of T-tubules was reduced in SPEG-knockout mice despite unaltered JPH2 levels, suggesting that SPEG phosphorylation of JPH2 is required to preserve T-tubule stability within cardiomyocytes, although a causal relationship remains to be established (117, 118). Moreover, SPEG was shown to phosphorylate RyR2 at a specific residue Ser2367, which surprisingly exerted an inhibitory effect on channel function in contrast to most other kinases (e.g., PKA, CaMKII) that enhance RyR2 channel activity (118–120).

#### 3.3.8. Posttranslational regulation of junctophilin.

The findings described above demonstrated that JPH2 might undergo posttranslational modifications (PTMs) that affects its functional activity within the JMC (116). These findings are in line with other observations that PTMs not only affect E-C coupling (10) but also the binding of regulatory proteins to JMC protein, such as RyR2 (121). In addition to SPEG phosphorylation, JPH2 may also be phosphorylated at Ser165. Woo et al. (122) demonstrated that the S165F variant, linked to hypertrophic cardiomyopathy in patients (see sect. 5.2), impairs protein kinase C (PKC)-mediated phosphorylation in myotubes expressing this variant. It remains to be established whether S165 phosphorylation occurs in vivo in the heart and whether this has any function consequences on E-C coupling.

Another PTM that modulates JPH2 is *S*-palmitoylation, a reversible attachment of a fatty acid chain to cysteine residues of the substrate protein. Jiang et al. (123) found that that JPH2 is *S*-palmitoylatable and that palmitoylation is essential for its SR/PM tethering function. *S*-palmitoylation of cysteine 678 was found to stabilize the JPH2 anchor into the ER membrane in COS-7 cells (123). Three other cysteine residues (Cys15, Cys29, and Cys328) were also modified by *S*-palmitoylation in in vitro experiments. *S*-palmitoylation was observed in native JPH2 in rat ventricular myocytes, where it helps JPH2 bind to lipid-raft domains (123). Sequence alignment of all four JPH isoforms revealed good conservation of the palmitoylatable Cys residues. The two Cys residues in the MORN-1 domain are conserved among all JPH isoforms, the Cys residue in MORN-8 domain in JPH1-JPH3, and the COOH-terminal Cys is conserved in JPH1, JPH2, and JPH4. These findings suggest that *S*-palmitoylation may also help other JPH isoforms stabilize the SR/ER-PM junctions.

Recent evidence also suggests that JPH2 might be oxidated (124). Oxidation mimicking substitutions of residues Cys678 and Met679 were found to augment the formation of JPH2 nuclear droplets, suggesting that oxidation or conditions associated with increased oxidative stress might affect the intranuclear assembly of JPH2 droplets, the significance of which remains largely unknown at this time (124). In addition, Phimister et al. (125) identified Cys101, Cys402, and Cys627 on JPH1 as highly reactive to thiols. Interestingly, the oxidation state of these residues was also heavily dependent on the conformational state of the associated RyR1 channel complex in a manner reported for a few hyperreactive thiols on RyR1 itself (126, 127). Thus it is likely that junctophilins are regulated by various PTM types, but the detailed mechanisms remain to be studied in detail.

## 4. CELLULAR FUNCTIONS OF JUNCTOPHILINS

### 4.1. Skeletal Muscle Cells

#### 4.1.1. JMC biogenesis in skeletal myofibers.

Junctophilin isoforms JPH1 and JPH2 play a role in the biogenesis of JMCs within skeletal myofibers. These subcellular structures are essential for the normal function of skeletal muscles, which participate in a variety of physiological functions, including breathing, swallowing, and body or eye movements. Skeletal muscles contract primarily in response to a voluntary stimulus controlled through motoneurons. When T-tubules become depolarized by an incoming action potential, the Cav1.1 channels undergo a conformational change resulting in a physical interaction with some RyR1 in clusters (128) (FIGURE 13), whereas lone RyR1 channels will be activated by Ca^2+^-induced Ca^2+^ release.

Representing the main Ca^2+^ store in striated muscle cells, the SR ensures the highly synchronized release of Ca^2+^ ions throughout the relatively voluminous cytosol surrounding the myofibrils. SR biogenesis starts with the formation of 30- to 60-nm wide ER tubules adjacent to the myofibrils (129). Next, branching tubules build a 3-D reticular ER network around the myofibrils (130). Finally, the myofiber-associated functionally mature SR engages in triad formation enabling rapid E-C coupling directly through JMC components connecting T-tubule invaginations locally at the sarcomeric A-I band interface as reviewed previously (131, 132). In skeletal muscles, the molecular components contributing to JMC biogenesis include JPH1, JPH2, caveolin 3, the skeletal muscle-specific isoform M-amphiphysin 2 [bridging integrator 1 (BIN1)], dysferlin, mitsugumins, and myotubulularin.

Electron microscopy has established the chronology of SR biogenesis during muscle differentiation in myofibers from mouse skeletal muscles (133). Maturation of RyR1 Ca^2+^ release channel clusters preceding the biogenesis of junctional SR complexes is accomplished at birth (133). The junctional SR develops its predominantly transverse orientation after birth. Between embryonic day (E)17 and E18, the final position of the triads in relation to both sides of the Z-line is established, ultimately coupling to single T-tubule between them (133). Interestingly, overexpression of SR proteins tagged with GFP variants in skeletal myotubes in culture showed that with a more differentiated SR organization, the immobile fraction of each junctional fusion protein of JPH1, Cav1.1-α1S, and RyR1 increased to over 50% specifically at junctional triads (86).

Notably T-tubule invaginations are formed only after SR biogenesis. In mouse embryos, short surface membrane-connected tubules can be observed at E15 during the invagination process in immature myotubes (133). Interestingly, at E16 mainly longitudinal tubules are prevalent, while newly formed tubules are connected with the surface through short transverse segments (133). Finally, the functionally mature, highly ordered transverse T-tubules complete their biogenesis at a high density 3 wk after birth in mouse skeletal muscles (133, 134). In summary, T-tubule biogenesis in skeletal myofibers is attributed molecularly to JPH1, JPH2, caveolin 3, BIN1, and dysferlin supporting the maturation of the electrically excitable endomembrane network in myofibers.

#### 4.1.2. Role of JPH1 in adult skeletal myofibers.

Highly differentiated organelle architectures and molecular compositions define the broad spectrum of skeletal muscle cells (myofibers) and their many specialized functions. The lumen of T-tubules in mature skeletal myofibers are ∼100 nm wide and continuous with the extracellular ion milieu, containing millimolar Ca^2+^ concentrations. Central for the precise contractile activation of multinucleated myofibers, a high density of T-tubules places clusters of membrane integral and associated JMC proteins near the smallest contractile units, the sarcomeres, a critical prerequisite for rapid transmission of action potential bursts, voluntary E-C control, and contractile activation of skeletal muscles (135). As myofibers are several millimeters to centimeters long and 50–100 μm in diameter, T-tubules provide specialized membrane structures in triads for activation of local Ca^2+^ release, physiologically connecting the sarcolemmal invaginations with the local SR Ca^2+^ stores. Hence, only through abundant T-tubule invaginations, striated muscles can electrically control their extensive cell volume (sarcoplasma) inside myofibers (135). The JPH1 interactions with the Cav1.1 channel have been discussed earlier (see sect. 3.3.1). Interestingly, JPH2 is also expressed in skeletal muscle while it is the predominant cardiac isoform (136). Importantly, direct physical interactions between the Cav1.1 and RyR1 channels in skeletal myofibers (FIGURE 13) support the transmission of voltage-dependent conformation changes into intracellular Ca^2+^ signals within 2 ms in skeletal muscles, which is in sharp contrast to 100 ms by diffusion-mediated Ca^2+^-induced Ca^2+^ release in cardiac muscles (137).

Visible as triad junction in sections of skeletal muscles by electron microscopy, two terminal cisternae of the junctional SR sandwich the T-tubule centrally between their membrane contacts (133). Hence, the uniform nanometric subspace distance in triads maintained inside JMCs by JPH1, JPH2, and RyR1 in mature myofibers represents a central prerequisite for the direct physical interaction between Cav1.1 and RyR1 channels reviewed earlier (see sect. 3.3.1) (93). In addition to JPHs, a host of proteins supports the equilibrium and repair of the triadic T-tubule/SR membrane assemblies. Conceptually, the protein assemblies at the specific localization at the interface between the T-tubule and junctional SR membranes are by definition collectively part of the JMC. Ultimately, thousands of triadic JMCs underlie the ultrarapid release of Ca^2+^ form the SR store, the upstroke of the myoplasmic Ca^2+^ transient, and myofiber single or sustained contractions (tetanus).

#### 4.1.3. Effects of JPH1 silencing and knockout.

Immunofluorescence experiments have demonstrated colocalization of Cav1.1 and RyR1 in subcellular clusters in vitro in C2C12 mouse skeletal myoblasts. Expression silencing of *JPH1* in HL-1 cells resulted in depressed Ca^2+^ transients and impaired Ca^2+^ influx (138). Moreover, *JPH1* expression silencing diminishes store-operated Ca^2+^ entry (SOCE) in vitro (93). Hence, *JPH1* silencing decreases the SR Ca^2+^ gradient and RyR1 Ca^2+^ release (93). Impairment of SOCE may involve disruption of Ca^2+^ release-activated Ca^2+^ modulator 1 (ORAI1) and stromal interaction molecule 1 (STIM1) (139). Following depletion of Ca^2+^ from the SR store, STIM1 oligimerization and interaction with ORAI1 activates SOCE at the surface membrane. Furthermore, transient receptor potential channel canonical type 3 (TRPC3) regulates RyR1 function (140). In mature skeletal myotubes, TRPC3 knockout has demonstrated impaired E-C coupling and RyR1 Ca^2+^ release despite preserved SR Ca^2+^ levels. Taken together, these in vitro results support a central role of JPH1 in skeletal muscle for JMC function to maintain E-C coupling, RyR1 Ca^2+^ release, and SOCE. SOCE dominates in vitro in immature myoblasts, particularly C2C12 cells (141). Interestingly, similar to findings during nonlphysiologic experimental conditions, SOCE plays a critical role in maintaining SR Ca^2+^ homeostasis during muscle fatigue (142). Hence, to elucidate the role of JPH1 in regulating SOCE will require future studies in in vivo models of intact, mature skeletal myofibers.

*Jph1* has been knocked out in mice, who die shortly after birth presumably from failure of milk suckling (141). Electron microscopy of skeletal muscles showed a reduction of triad junctions and abnormal SR structures (141). Additionally, muscle preparations from *Jph1*-deficient mice develop less contractile force (141). Taken together, while both JPH1 and JPH2 are abundantly expressed in skeletal muscles, *Jph1* knockout disrupts the JMC number, triad architecture, rapid E-C coupling, and the physiological function of skeletal muscles to an extent that is not compatible with life.

### 4.2. Cardiac Muscle Cells

#### 4.2.1. Role of JPH2 in cardiomyocyte T-tubule development.

**FIGURE 15.**
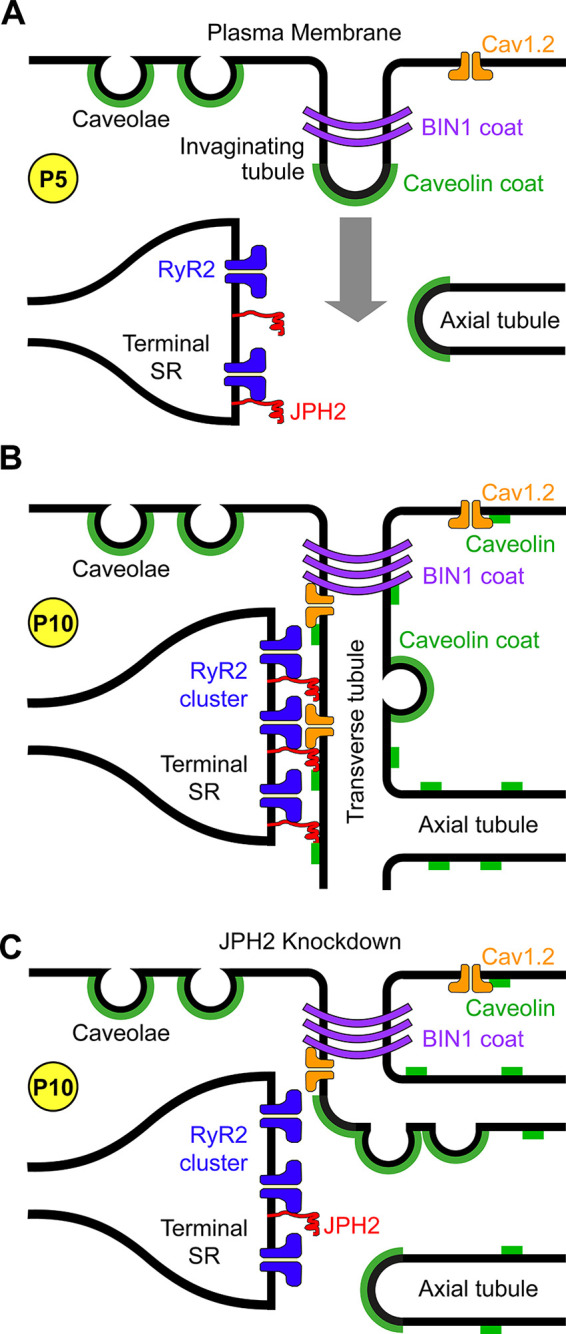
Membrane tubulation processes in cardiomyocytes. *A*: possible mechanism of invaginating T-tubule development at postnatal day (P)5. Caveolin 3 is indicated as coat protein of caveolae (green), bridging integrator 1 (BIN1) as membrane folding coat near the cell surface at the rudimentary tubule (lilac), as well as junctophilin 2 (JPH2) (red) and ryanodine receptor 2 (RyR2) (blue) in the terminal sarcoplasmic reticulum (SR). *B*: from P10 and later mature T-tubules with SR membrane contacts have formed. Increased JPH2 and RyR2 expression and coclustering in the terminal SR is tightly coordinated with Cav1.2 clustering in JMCs at T-tubules. Note the nonlcaveolar caveolin 3 complexes (green rectangles) and interactions with Bin1, Cav1.2, and JPH2. Axial tubules are connected to T-tubules in a mature cell-wide network. *C*: acute shRNA-mediated JPH2 knockdown disrupts anchoring of developing T-tubules to the terminal SR and intracellular network maturation. Proliferation of axial tubules and irregular membrane structures is increased and overall similar to observations in heart disease. Modified from with permission from Beavers et al. (145).

In cardiomyocytes, E-C coupling depends on Ca^2+^ influx-dependent activation of RyR2 (FIGURE 14). In mammalian ventricular cardiomyocytes, the T-tubule system is absent or only rudimentary at birth (95, 143, 144). Normal T-tubule development involves JPH2, caveolin-3, and BIN1 (FIGURE 15*A*). For example, while neonatal rat cardiomyocytes lack T-tubules at P5 when the cells are immature, with increasing size by postnatal day (P)20 they develop a cell-wide T-tubule system indistinguishable from that of adult cardiomyocytes (95). Associated with upregulation of postnatal JPH2 protein expression, T-tubule maturation results in membrane invaginations enriched with ∼80% of the total cellular pool of Cav1.2 channels (FIGURE 15*B*) (143, 146). Hence, fundamentally different from skeletal myofibers, these molecular features provide the rapid transmembrane Ca^2+^ influx for initiation of E-C coupling through activation of nearby RyR2 channel clusters in the junctional SR by the end of the first month of life. Taken together, both postnatal maturation and precise sarcolemmal T-tubule positioning at Z-disks opposite the Ca^2+^ release sites of the terminal SR store in JMCs are required for beat-to-beat Ca^2+^ induced Ca^2+^ release in adult ventricular cardiomyocytes.

Interestingly, the muscle-specific membrane-integral protein caveolin-3, abundantly clustering in the T-tubule network of adult ventricular cardiomyocytes (112), colocalizes with the junctional SR protein JPH2 throughout development mainly at the surface membrane but also at sparse primitive T-tubules in neonatal cardiomyocytes (95). However, the profound increase in E-C coupling efficiency through rapid local Ca^2+^ signaling between the Cav1.2 and RyR2 channel clusters occurs only when JPH2 and RyR2 become colocalized (FIGURE 15*B*), with a slight delay in neonatal cardiomyocytes following T-tubule maturation (95). For detailed molecular interactions between JPH2, caveolin-3, Cav1.2, and RyR2, please refer to sect. 3.3. Hence, JPH2 and its interactions with JMC-specific proteins in T-tubules play a significant role in the postnatal maturation sprint and the maintenance of ventricular cardiomyocytes.

Other proteins are known to contribute to T-tubule biogenesis and membrane folding. The muscle-specific isoform of bridging integrator 1 (BIN1; also known as M-amphiphysin-2) has been shown to induce actin-stabilized inner membrane folds each within T-tubules and outside T-tubules to anchor them to Z-disks mainly at the cortical region of cardiomyocytes (FIGURE 15*A*) (147). BIN1 can induce Cav1.1-enriched tubule invaginations in skeletal C2C12 myotubes and interacts and colocalizes with Cav1.2 in ventricular cardiomyocyte T-tubules (148, 149). Only a splice-isoform containing a phosphoinositide-binding motif, pBIN1 + 13 + 17, can mediate the membrane shaping of luminal T-tubule folds (147, 150). Similar to JPH2, neonatal cardiomyocytes exhibit a very low expression of BIN1 isoforms (147). Immunoblotting based on an antibody against the phosphoinositide-binding motif has recently demonstrated the location of pBIN1 both in cardiac and skeletal muscle (151). While pBIN1 and uBIN1 are differentially localized in neonatal cardiomyocytes, overexpression of both fluorophore-tagged isoforms in cultured neonatal cardiomyocytes has resulted in induction of tubulation and the cooperative binding of both proteins to membrane tubules at Z-disks (151). Distinct BIN1 isoforms may contribute to this T-tubule membrane folding and together with JPH2 support the postnatal tubulation spring and tubular Cav1.2 enrichment linked through JPH2 binding to RyR2 Ca^2+^ release sites.

#### 4.2.2. Cardiomyocyte and T-tubule network maturation.

Studies in embryonic cardiomyocytes correlate further with studies in differentiated cardiomyocytes. Several investigators have demonstrated that JPH2, a CAV3-interacting protein, has a central role in the development and maintenance of mature T-tubules and JMCs after birth (111, 152, 153). With the full maturation of T-tubules and rising postnatal protein expression, JPH2 colocalizes increasingly with Cav1.2 channel clusters, which coincides further with increased RyR2 expression and additional gradual colocalization in JMCs (95, 154). Taken together, the maturation of functionally competent, deep intracellular Ca^2+^ release sites require distinctive maturation steps at the level of JMCs and T-tubules after birth. T-tubules in both in atrial and ventricular cardiomyocytes are further connected to deep intracellular axial (longitudinal) tubules, effectively forming cell type-specific excitable endomembrane networks (98, 155).

Acute RNAi-mediated knockdown of *Jph2* in cultured adult rodent cardiomyocytes resulted in cell-wide T-tubule disorganization (156). Scanning ion conductance microscopy of ventricular myocytes from patients with cardiomyopathy revealed additional changes in the surface structure of T-tubules, including flattening and loss of Z-groove definition (156). Short-hairpin RNA (shRNA)-mediated knockdown of *JPH2* caused T-tubule disruption while the fraction of axial (longitudinal) tubules was increased (FIGURE 15*C*) (154, 157). *Jph2* expression silencing in vitro in cardiac cells resulted in impaired CICR and less frequent generation of spontaneous Ca^2+^ transients, as well as decreased Ca^2+^ transient amplitude (138). The latter findings were not caused by expression changes of any Ca^2+^ transporter proteins implicating further an altered function of RyR2 rather than altered protein expression. Decreased levels of JPH2 in mouse hearts were associated with defective postnatal T-tubule maturation by P8 (158). In adult mice, inducible *Jph2* knockdown resulted in an increased Ca^2+^ spark frequency and an impaired E-C coupling gain consistent with RyR2 channel dysfunction (6). Coimmunoprecipitation of RyR2 confirmed its binding to JPH2, further suggesting a role in regulation of RyR2 channel function. Specifically, direct binding of JPH2 to RyR2 stabilizes the channel closed state, whereas decreased JPH2 expression resulted in increased RyR2 Ca^2+^ leak with blunting of E-C coupling. Cav1.2 also coimmunoprecipitates with BIN1, which anchors microtubules at JMCs facilitating the trafficking of this channel to Ca^2+^ release units (149). While BIN1 levels remained unchanged, cardiac-specific shRNA-mediated knockdown of *Jph2* in mice prevented the formation of mature T-tubules in the first weeks after birth (152, 153). Disruption following acute *Jph2* knockdown preferentially decreased transverse components suggesting that axial tubules may depend less on JPH2 for postnatal maturation in ventricular cardiomyocytes (153). In summary, these studies suggest that the maintenance of T-tubules in ventricular cardiomyocytes is a process coordinated through multiple and distinct protein interactions where JPH2 supports membrane invagination, tubule elongation and maintenance, protein trafficking, and JMCs.

Given that JPH2 plays a central role in the development and stability of cardiomyocyte endomembrane ultrastructure, complementary JPH2 overexpression studies are important. Mice overexpressing JPH2 showed accelerated T-tubule maturation by P8 (158). In mice engineered for cardiomyocyte-restricted JPH2 overexpression, T-tubule maturation occurred earlier in ventricular myocytes (152). JPH2 overexpression using the same mouse model promoted substantially larger JMCs with polyadic junctions and larger Ca^2+^ transients in adult atrial cardiomyocytes demonstrated recently by stimulated emission depletion (STED) microscopy and electron tomography (152). The latter polyadic JMC augmentation occurred despite ∼70% lower JPH2 protein levels in atrial compared with ventricular tissue indicating a large margin of atrial JMC plasticity raising the potential for therapeutic augmentation in heart disease (82). Recently, JPH2 overexpression prevented the remodeling of JPH2, caveolin-3, and T-tubules in cultured ventricular cardiomyocytes, while the number of functional Cav1.2 channels was increased (113). Taken together, these studies suggest that JPH2 supports both endomembrane structural maturation as well as protein organization in JMCs deep inside cardiomyocytes.

### 4.3. Smooth Muscle Cells

As introduced in sect. 3.3.3, contraction of smooth muscle cells is primarily regulated by an increase in cytosolic Ca^2+^ concentration (159). Similar to cardiac myocytes, membrane depolarization triggered CICR involving LTCC and RyRs in smooth muscle (160). In addition, extracellular ligands can bind to PM-localized receptors that generate 1,4,5-trisphosphate (IP_3_), which diffuses across the cytosol and activates intracellular Ca^2+^ release channels known as IP_3_ receptors (IP_3_R) (160). Due to the loose coupling of LTCC and RyRs in smooth muscle, LTCC opening does not always trigger RyR activation (161). Furthermore, smooth muscle cells lack T-tubules that enhance the ER-PM contact area. Therefore, JMCs may not be as critical for E-C coupling in smooth muscle.

However, junctophilins do appear to facilitate the coupling between RyRs and large conductance Ca^2+^-activated K^+^ channels, commonly called BK or big potassium channels. JPH2 is the most abundant JPH isotype in vascular smooth muscle (106). *Jph2* knockdown causes increased vascular smooth muscle contractions (106, 162). Ca^2+^ sparks from spontaneous opening of RyRs activate nearby BK channels and generate an outward K^+^ current (163). Moreover, the outward current activated by spontaneous Ca^2+^ sparks is reduced when JPH2 is knocked down, consistent with a role for JPH2 in coupling RyRs and BK channels (106, 162). Thus JPH2 plays an important role in maintaining vascular smooth muscle resting tone by coupling RyRs to BK channels. In addition, JPH2 expression was detected in mouse stomach and lung (2), which contain smooth muscle.

### 4.4. Neurons

#### 4.4.1. Roles of JPH3 and JPH4 in the central nervous system.

In the brain, JPH3 and JPH4 are primarily expressed in neurons. JPH3/4 mediate central neuronal functions such as balance and motor control through maintenance of intracellular Ca^2+^ signaling. Indeed, at 3 mo of age *Jph3* knockout mice develop an impaired balance and motor coordination without significant defects in brain morphology or molecular signaling (164). *Jph3* knockout mice showed no overt disruption in neuronal tissue architecture. Furthermore, *Jph3* knockout did not result in apparent action potential changes in Purkinje neurons while synaptic dysfunction was excluded in brain tissue (165). However, by 6 and 9 mo of age, *Jph3* biallelic and monoallelic knockout in mice developed progressive defects in balance, coordination, neuromuscular strength, with a gene dosage-dependent defect (14). Moreover, *Jph3* knockout mice develop some behavioral abnormalities (164, 165). Compared spatially in brain slices to JPH3, JPH4 localizes to discrete areas, while *Jph4* knockout has no discernible neurological phenotype (11, 108). The latter findings suggest that JPH4 is not required for brain development, raising the possibility that this isoform may be redundant. The electrophysiological effects of JPH3 have only been studied in a few cell types, and the effects on striatal cells, implicated in the pathogenesis of Huntington Disease-Like 2 (HDL2) (see sect. 5.3), have not been studied, for instance. Therefore, additional studies on the roles of JPH3 and JPH4 in cell types linked to disease phenotypes are warranted.

Neurons from *Jph3/4* double knockout mice demonstrated disrupted intracellular Ca^2+^ signaling (11). In hippocampal neurons, a disrupted communication between plasmalemmal Ca^2+^ entry via NMDA glutamate receptors, intracellular RyR channels, and small-conductance Ca^2+^-activated potassium (SK) channels was observed (11). Under physiologic conditions, proper signaling between these plasmalemmal and intracellular channels is necessary for local control of intracellular Ca^2+^ signaling in hippocampal neurons (11). In addition to the hippocampal neurons, *Jph3/4* double knockout mice develop impaired Ca^2+^ signaling in Purkinje cells between P/Q-type voltage-gated Ca^2+^ channels and RyR channels further disrupting the SK channel-mediated afterhyperpolarization (166). *Jph3/4* double knockout in mice demonstrated a decreased exploratory activity and memory, as well as premature death of young mice at 5 wk of age (11). These studies point to defective Ca^2+^ storage based on leaky RyR channels associated with neuronal JPH deficiency. While the precise roles of RyR isoforms in neuronal tissue are understudied, early studies have viewed RyR3 as canonical major isoform while RyR1 and RyR2 are also expressed (11, 167). Short hairpin-mediated knockdown of *Jph3* and *Jph4* in rat CA1 pyramidal cells led to dissociation of a Cav1.3-RyR2-KCa3.1 complex, leading to reduced Ca^2+^-dependent slow afterhyperpolarizations (168). These findings suggest that both JPH isoforms maintain a macromolecular channel complex that allows two Ca^2+^ sources to act in tandem to define the activation properties of KCa3.1 channels in neurons.

While the roles of specific RyR isoforms for ER Ca^2+^ release have not been clarified in the context of the many different neuron subtypes in the brain, one hypothesis has been that RyR isoforms provide cell type-specific functions (169, 170). Further evidence suggested that neuronal LTCCs may directly couple with RyR1 channels analogous to direct physical RyR1 opening in skeletal muscle (FIGURE 13). Interestingly, heterozygous knockin of the disease-causing RyR1-I4895T variant in mice causes impaired voltage-dependent Ca^2+^ release from intracellular stores in addition to myopathy in skeletal muscles (171). In the brain, RyR1 is expressed abundantly in the cerebellum and RyR2 in the cerebrum. Interestingly, heterozygous knockin mice with the R2474S variant in RyR2 developed seizures and altered Ca^2+^ signal bursts in hippocampal neurons (172). *Jph3* hemizygous and null knockout mice exhibiting abnormal motor function were recently compared with Huntington disease-like 2 and Huntington disease mouse models, extending mechanisms both through a multifactorial toxic gain-of-function of *Jph3* RNA and a loss of JPH3 expression (165).

*Jph3/4* double knockout mice exhibited altered synaptic plasticity in the corticostriatal circuits and irregular methamphetamine-induced behavioral sensitization (173). This was associated with aberrant CaMKII autophosphorylation and increased calcineurin activity in the striatum of *Jph3/4* knockout mice and may account for lack of methamphetamine-induced behavioral sensitization. *Drosophila* RNAi-based screening showed that knockdown of retinophilin protects axons from degeneration exposed to taxol, whereas retinophilin knockdown delays degeneration in severed olfactory axons (174), while a functional interaction of JPH2 with the Ca^2+^-activated small-conductance potassium channel 2 (SK2) in mouse cardiomyocytes (175) was recently associated with the MORN domains in HEK293 overexpression studies (176). The SK2 channels play important roles in synaptic plasticity, learning, and memory, while alterations in SK2 expression and regulation may contribute to brain disorders including Alzheimer’s and Parkinson’s disease (177). These studies raise the possibility that uncoupling of voltage-sensitive Ca^2+^ channels from RyRs and disruption of Ca^2+^-activated SK2 channel functions in neurons of *Jph3/4*-deficient mice may affect the complex multifactorial cross talk of Ca^2+^ signaling in JMCs of specific neuron types.

#### 4.4.2. Roles of JPH3/4 in peripheral neurons.

There is also emerging evidence that JPH plays a role in the peripheral nervous system. In *C. elegans*, the single JPH isoform was shown to localize to discrete membrane contact sites in neurons and muscles (178). In neurons, JPH colocalized within the membrane contact site protein Extended SYnaptoTagmin 2 (ESYT-2) in the soma and was found near presynaptic release sites suggesting a role in neuromuscular synaptic transmission (178). In mammals, in vivo knockdown of *Jph4* in the dorsal root ganglion sensory neurons was shown to significantly attenuate experimentally induced inflammatory pain in rats (178). Using fluorescence imaging, proximity ligation, superresolution microscopy, and in vitro and in vivo gene knockdown, Hogea et al. (12) showed that JPH4 is essential for the formation of the SOCE complex at the ER-PM junctions in rat somatosensory neurons. Thus junctional nanodomain Ca^2+^ signaling maintained by JPH4 is an important contributor to the inflammatory pain mechanisms (12).

### 4.5. Other Cell Types

While JPH3/4 have been mainly studied in the context of neurons, other electrically excitable cells may depend on these JPH isoforms. For example, in pancreatic beta cells JPH3 is required for glucose-dependent insulin secretion (179). Moreover, JPH4 is expressed in JMCs of T cells, regulating their intracellular Ca^2+^ signaling (24). While a thorough review of all excitable cell types is beyond the focus of this review, future cell biology studies will without doubt uncover additional JPH roles and extend these to the vast number of cell type-specific functions.

## 5. INHERITED DISEASES CAUSED BY JUNCTOPHILIN GENE VARIANTS

Various types of deficits in members of the junctophilin gene family have been associated with several inherited disorders. Autosomal-dominant and recessive variants in *JPH2* have been linked to hypertrophic and dilated cardiomyopathy, respectively (180, 181). *JPH1* has been revealed as a modifier gene of a hereditary motor and sensory neuropathy known as Charcot-Marie-Tooth disease (182). Moreover, microsatellite expansions in the *JPH3* gene have been linked to a progressive movement disorder known as Huntington Disease-Like 2 (17). The following sections will provide an in-depth review of the impact of genetic variants in each of the *JPH* genes on human disease phenotypes.

### 5.1. JPH1 Variants and Charcot-Marie-Tooth Disease

Charcot-Marie-Tooth (CMT) disease is a group of hereditary motor and sensory neuropathies that affects an estimated 128,000 individuals in the United States alone with an incidence of ∼1:2,500 people. CMT primarily affects peripheral nerves in the arms and legs resulting in muscle atrophy and weakness typically noticeable in adolescence of early adulthood. Nearly all cases of CMT are inherited in one of three distinct patterns: autosomal dominant, autosomal recessive, and X-linked (183). There are many different types of CMT, which may share some symptoms but vary by pattern of inheritance, age of onset, and whether the axon or myelin sheath is involved. CMT type 2 (CMT2) results from abnormalities in the axon of the peripheral neuron rather than the myelin sheath and is less common that CMT1 (184). This autosomal-dominant disorder has more than a dozen subtypes, with each subtype being associated with mutations in specific genes.

Autosomal-dominant variants in the ganglioside-induced differentiation-associated protein 1 (*GDAP1*) gene cause CMT type 2 K (CMT2K). Due to its position near the *GDAP1* gene on chromosome 8q21.1, *JPH1* was hypothesized to be a disease-modifier gene in individuals with CMT2K. *JPH1* sequence analysis in 24 carriers of *GDAP1* variants revealed 2 variants: R213P in one patient and D624H in one patient and three affected siblings from another family (182). The R213P substitution is located in the joining region between the first six and last two MORN motifs. This patient exhibited a moderately severe phenotype, with onset of walking difficulties in her early teens, amyotrophy of the legs, and pes cavus with claw toes. The patient’s father, who only carried the *GDAP1* variant, had a very mild CMT phenotype, while the patient’s mother, who only carried the JPH1 variant, was unaffected, consistent with a disease-modifier role of JPH1 in CMT.

Wild-type JPH1, but not R213P mutant JPH1, was able to normalize store-operated calcium release (SOCE) in GDAP1-deficient neural crest cells. Further cellular studies revealed that JPH1 colocalizes with stromal interaction molecule 1 (STIM1), which is the activator of SOCE during ER Ca^2+^ release. In contrast, the R213P mutant JPH1 exhibited impaired oligomerization with STIM1 and showed a higher colocalization with mitochondria in GDAP1-deficient cells (182). The presence of GDAP1 and JPH1 variants together resulted in significantly impaired SOCE, causing an increase in cytosolic Ca^2+^ levels, which might be associated with cellular toxicity. In contrast, when GDAP1 was added, normal colocalization signal patterns in neural crest cells were restored suggesting that JPH1 activity is GDAP1 dependent. However, GDAP1 does not appear to physically interact with JPH1; therefore, how GDAP1 is able to modify JPH1 expression patterns remains unknown (182).

### 5.2. JPH2 Variants and Inherited Cardiomyopathies

#### 5.2.1. Genetic variants in JPH2 linked to human disease.

Cardiomyopathies are a common cause of morbidity and mortality worldwide. Hereditary cardiomyopathies are classified according to their clinical manifestations as hypertrophic cardiomyopathy (HCM), dilated cardiomyopathy (DCM), and arrhythmogenic cardiomyopathy (185). Other forms of cardiomyopathies that are less common include restrictive cardiomyopathy, left ventricular nonlcompaction cardiomyopathy (LVNC), and amyloid cardiomyopathy. Mutations in the *JPH2* gene have been associated with several types of cardiomyopathy. The first genetic variants in *JPH2* were identified in patients with HCM, an inherited disorder characterized by cardiac hypertrophy, a preserved or increased ejection fraction, and in ∼25% of the patients also left ventricular outflow tract obstruction (LVOTO) (180). HCM is a major cause of sudden cardiac death in the young as a result of ventricular fibrillation (186). Moreover, atrial arrhythmias including atrial fibrillation are also seen in a subset of HCM patients (187).

HCM affects ∼1 in 500 in the general population and can be caused by genetic variants in a variety of genes. A systematic assessment of clinical genetic and experimental data using a scoring matrix revealed that the majority of genes previously reported as causative of HCM had limited to no evidence of disease association (188). Out of 33 genes classified for HCM, 8 were classified as definitive, 3 as moderate, 16 as limited, and 6 as no evidence. *JPH2* ranked in the second-highest category (moderate) based on evidence that includes either segregation evidence, reported de novo variants, and some experimental evidence (188). In addition, examination of the public repository ClinVar (189) revealed that *JPH2* ranked among the “definitive HCM and syndrome genes” and the “moderate evidence genes” (188).

As of October 2021, 336 *JPH2* variants have been reported in the ClinVar database (189), as retrieved by the gnomAD browser (version 2.1) (190). Of these, 109 are synonymous substitutions that do not change the protein sequence. While is it possible that a synonymous variant can disrupt transcription, splicing, cotranslational folding, or mRNA stability, relatively little remains known about this type of variants and current computational approaches are not able to predict their potential impact (191). Of the remaining 227 non-synonymous variants, 12 ClinVar variants are classified as putative loss-of-function (pLOF) variants that include nonsense, frameshift, and splice site alterations (Trp64Ter, Gln142Ter, Ala181ProfsTer125, Gln364Ter, Gln428Ter, Gln493Ter, Gln549Ter, Gln564Ter, Glu641Ter, Lys651RfsTer30, Arg655Alafs57, and c2006_2010 + 1dup; see TABLE 2). These variants are all predicted to truncate the JPH2 protein and have a severe loss-of-function phenotype. Jones et al. (181) recently reported one of these pLOF variant (Glu641Ter) in 2 Iranian families. One proband who was homozygous for this variant was diagnosed at 20 mo of age with DCM. By the age of 4.5 yr, the patient suffered from severe systolic heart failure and cardiac conduction disease. The child died at 5 yr of age while awaiting transplant. The second proband was identified in another consanguineous family. While his DNA was not obtained, he died at 2.5 yr of age from cardiac failure, and both parents were heterozygous for the E641* variant (181). Jones et al. (181) described an additional nine pLOF variants in whole exome sequencing (WES) cohorts of American and Iranian patients. However, due to a lack of clinical information for those variant carriers, it remains unknown whether any suffered from cardiomyopathy and they were not included in TABLE 2.

**Table 2.** Genetic variants in JPH2 with predicted loss-of-function and/or cardiac disease association, as listed in the ClinVar database

Variant (DNA)	Variant (Protein)	Inh. Mode	Variant Type	Sex and Age of Diagnosis	(Suspected) Structural Heart Disease	(Suspected) Arrhythmias	Reference and/or ClinVar No.
	p.Gly21Ter		VUS, pLOF				gnomAD2.1
	p.Trp54Ter		US, pLOF				gnomAD2.1
c.163C>G	p.Pro55Ala		VUS	M86	HCM, diastolic dysfunction, atrial enlargement, amyloidosis	None described	(192)
c.191G>A	p.Trp64Ter		LP, pLOF	N/A	HCM		829986
c.253G>A	p.Glu85Lys	AD	VUS	M51, M65, M49, M20, M39, M37, M12	DCM phenotype with varying degrees of LV dilatation and systolic dysfunction (5/7), RV dilatation/dysfunction (5/7), diastolic dysfunction (3/7), severe mitral/tricuspid regurgitation (1/7), severe pulmonary hypertension (1/7), LVNC (2/7)	3rd degree AVB (2/7), 1st degree AVB (1/7), bradycardia (3/7), LBBB or RBBB (2/7), pathological q waves in precordial leads (2/7)	(193)
c.301A>C	p.Ser101Arg	AD	P	M27	HCM phenotype	None described	(180)
c.406G>A	p.Gly136Ser		VUS	N/A	HCM		(194)
c.421T>C	p.Tyr141His	AD	VUS	M24	HCM phenotype with LVOTO	None described	(180)
c.424G>T	p.Gln142Ter		VUS, pLOF	N/A	Unknown		488956
	p.Val143AlafsTer160		VUS, pLOF				gnomAD2.1
c.482C>A	p.Thr161Lys	AD	LP, VUS	7 families, 26 patients, M (range: 8–62), F (range: 8–80)	HCM phenotype (20/26), systolic heart failure (3/26), LVOTO (2/26),	Conduction abnormalities (14/26), AF (9/26), SVT (5/26), VT (6/26)	155800, (195)
	p.Ser162ArgfsTer144		VUS, pLOF				gnomAD2.1
c.493_514delinsC	p.Ser165Asn172delinsHis		VUS	N/A	HCM		(194)
c.494C>T	p.Ser165Phe	AD	P	F30	HCM phenotype LVOTO	None described	(180)
c.505G>A	p.Glu169Lys		VUS	M (at birth), M22, M (unknown)	HCM with LVOTO, HCM (2 patients)	AF, SSS, QT prolongation (1 patient), paroxysmal AF and SA block (1 patient), paroxysmal SVT (1 patient)	(96, 187)
c.541del	p.Ala181Pro-fs*125		VUS, pLOF	N/A	DCM		636877
	p.Ser182Ter		VUS, pLOF				gnomAD2.1
c.559G>A	p.Ala189Thr		VUS	M22	HCM phenotype with mild bilateral atrial dilatation (1 patient); DCM (1 patient), ARVC (1 patient), HCM (1 patient)	Arrhythmia and SCD (1 patient)	180593, (196)
c.620A>G	p.Asn207Ser		VUS	N/A	HCM		(194)
	p.Lys229AlafsTer70		VUS, pLOF				gnomAD2.1
c.692G>A	p.Arg231Gln		VUS	N/A	HCM (1 patient), inborn genetic disease (1 patient)		(194, 197)
c.709A>G	p.Thr237Ala	AD (compound HZ with I414L)*	VUS	F0 (8 mo)	DCM features with LV dilatation, slight LVNC, several MV regurgitations, reduced EF, died at 8 mo of age due to acute HF		(198)
c.723C>G	p.Ser241Arg		VUS	N/A	DCM (1 patient), HCM (1 patient)	Paroxysmal familial ventricular fibrillation (1 patient)	155801, (195)

	p.Glu271Ter		VUS, pLOF				gnomAD2.1
c.1013A>G	p.Glu338Gly		VUS	F30	HCM	Sudden cardiac death	(199)
c.1033G>C	p.Val345Leu		VUS	M67, N/A	HCM	Drug-induced LQTS (1 patient)	(194, 200)
c.1083G>A	p.Lys361Asn		VUS	N/A	HCM		(194)
c.1090C>T	p.Gln364Ter		VUS, pLOF	N/A	Unknown (2 patients)		201800
	p.Gln382Ter		VUS, pLOF				gnomAD2.1
c.1213G>T	p.Ala405Ser	De novo	VUS	M16, N/A (2×)	HCM features with basal septal hypertrophy, LVOTO (1 patient); HCM (1 patient), unknown (1 patient)	Left anterior fascicular block, ST-segment and T-wave abnormalities, prolonged QTc (1 patient)	372724, (96, 201, 202)
c.1240A>C	p.Ile414Leu	AD (compound HZ with T237A)*	VUS	F0 (8 mo)	DCM features with LV dilatation, several MV regurgitation, reduced EF, died at 8 mo of age due to acute HF		(198)
c.1282C>T	p.Gln428Ter	AR	VUS, pLOF	F3	Severe DCM requiring heart transplant at age 4		(203)
c.1357C>T	p.P453Ser			N/A	HCM (1 patient), CV phenotype (1 patient)		264361, (194)
	p.Pro466LeufsTer30		VUS, pLOF				gnomAD2.1
c.1399C>G	p.Arg467Gly		VUS	N/A	HCM		569250, (194)
	p.Glu476Ter		VUS, pLOF				gnomAD2.1
c.1477C>T	p.Gln493Ter		VUS, pLOF	N/A	HCM		863336
c.1513G>A	p.Gly505STer (reclassified as common variant)	AD	VUS	M14, F40, F33, F3, M1	HCM features with LVH (1 patient), HCM with sigmoid septum (1 patient), hyperdynamic LV motion (1 patient), symmetric hypertrophy IVS (1 patient)	Deep Q wave, ST-T changes (1 patient), sudden cardiac death (1 patient)	(204–208)

c.1645C>T	p.Gln549Ter		VUS, pLOF	N/A	HCM		947259
	p.Tyr563Ter		VUS, pLOF				gnomAD2.1
c.1690C>T	p.Gln564Ter		VUS, pLOF	N/A	DCM		636998
c.1778A>G	p.Glu593Gly		VUS	N/A	HCM		(194)
c.1790C>G	p.Ser597Trp		VUS	N/A	HCM		(194)
c.1920dupT	p.Glu641Ter	AD, AR	LP, pLOF	2 families: family 1: M0 (10 mo), M (8 weeks); family 2: M0 (5 mo)	DCM, severe LV systolic dysfunction, HF (2 patients); 1 sudden death; 1 Epstein anomaly and progressive LV failure following surgery	PR prolongation, conduction delay, T-wave abnormalities	(181)
	p.Arg643Ter		VUS, pLOF				gnomAD2.1
c.1952del	p.Lys651Arg-fs*30		VUS, pLOF				809249
c. 1962dup	p.Arg655Ala-fs*57		VUS, pLOF	N/A	HCM		1004247
c.2006_2010 + 1dup	Splice donor		VUS, pLOF				1034663
c.2011-1G>T	Splicing change		VUS	N/A	HCM		(209)

This table includes junctophilin 2 (*JPH2*) variants listed in the ClinVar database that are *1*) classified in ClinVar as pathogenic/likely pathogenic (P/LP), *2*) classified in ClinVar and/or gnomAD2.1 as a variant of uncertain significance (VUS) with a predicted loss-of-function phenotype (pLOF), or *3*) variants characterized in published papers. AD, autosomal-dominant; AF, atrial fibrillation; AR, autosomal-recessive; ARVC, arrhythmogenic right ventricular cardiomyopathy; AVB, atrioventricular block; CV, cardiovascular; DCM, dilated cardiomyopathy; del, deletion; dup, duplication; EF, ejection fraction; F, female; fs, frameshift; HCM, hypertrophic cardiomyopathy; HF, heart failure; HZ, heterozygous; ins, insertion; IVS, interventricular septum; LQTS, long QT syndrome, LV, left ventricle; LVH, left ventricular hypertrophy; LVNC, left ventricular nonlcompaction; LVOTO; left ventricular outflow tract obstruction; M, male; MV, mitral valve; RV, right ventricle; SCD, sudden cardiac death; SSS, sick sinus syndrome; SVT, supraventricular tachycardia; ter, termination; VT, ventricular tachycardia; US, unknown significance.

**FIGURE 16.**
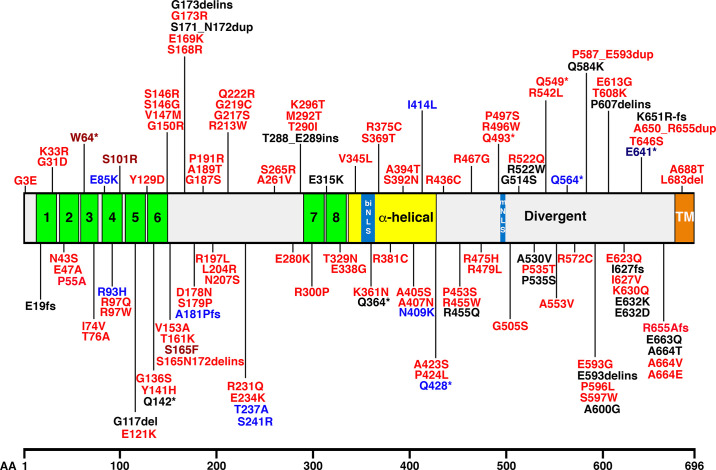
Distribution of junctophilin 2 (JPH2) variants in ClinVar superimposed onto JPH2 protein domains. The bar in the *middle* of the figure represents the human JPH2 protein; functional domains are marked as follows: number green bars represent membrane occupation and recognition nexus (MORN) domains, the yellow box represents the alpha-helical domain, the divergent domain is white, and TM marks the transmembrane domain. The numbers represent amino acids residues; the letters represent amino acid identifiers. biNLS, biphasic nuclear localization signal; mNLS, monopartite nuclear localization signal; del, deletion; dup, duplication; fs, frameshift; ins, insertion. *Stop codon. Red marks hypertrophic cardiomyopathy-linked variants, and dark red indicates LP/P variants (W64*, S101R, S165F). Blue marks dilated cardiomyopathy-linked variants, dark blue indicates LP/P variant (E641*). Black marks variants with an unknown disease association.

The classification of genetic variants, based on the American College of Medical Genetics guidelines, is usually based on a five-tiered scheme that considers the quantity and quality of evidence needed to classify a given variant as pathogenic, likely pathogenic, a variant of uncertain significance (VUS), likely benign, or benign (210). If the classification of the variant is deemed a VUS, it means that, at the time of interpretation, there was insufficient evidence to determine if the variant is related to a specific disease or not. ClinVar revealed that only 4 *JPH2* variants are currently classified as pathogenic or likely pathogenic (Trp64Ter, Ser101Arg, Ser165Asn, and Glu641Ter) (180) (FIGURE 16). Two of these four are pLOF variants, which means that the other pLOF variants were classified as VUS, which is surprising based on the predicted effects on protein structure. An additional 179 variants in ClinVar were also classified as VUS due to a lack of definitive evidence linking the variant to HCM or another type of disease. An additional 12 pLOF variants have been reported in the gnomAD2.1 database, but for those no clinical information was available (TABLE 2). These are all rare variants that are predicted to impair JPH2 protein expression or function.

The T161K variant was originally reported as a VUS (ClinVar #155800). Subsequently, a paper reported segregation of this *JPH2* variant with HCM in seven families (195), which allowed for reclassification of this variant from VUS to pathogenic (210). At present, however, this variant is listed as “disputed” in ClinVar, with one testing company listed it as VUS and one as LP. It is anticipated that the ClinVar classification will change to P in the near future. On the other hand, variant Gly505Ser was initially described as a disease-associated *JPH2* variant (204). However, as a result of further genetic analysis that revealed a global minor allele frequency of 0.015, this variant has been downgraded from VUS to benign/likely benign (205–208). Another study reported the Gly505Ser variant in 2.9% (100/3,426) of South Asian chromosomes (190).

#### 5.2.2. Clinical manifestations of JPH2 variants.

For the majority of *JPH2* variants listed in ClinVar, clinical information except for the suspected diagnosis is not available (FIGURE 16). Most of the variants included in TABLE 2 have been linked to HCM, while some variants were clearly linked to DCM, and for other variants insufficient details were available. For 27 variants, however, more detailed clinical descriptions have been reported in published papers. The first study to describe *JPH2* variants linked to human disease described clear echocardiographic features for HCM in North American patients of Caucasian descent (180). LVOTO was reported in patients with the Tyr141His, Thr161Lys, Ser165Phe, Glu169Lys, and Ala405Ser variants (96, 180, 187, 195, 201). Among other ventricular phenotypes, left ventricular diastolic dysfunction has been reported in association with two HCM-linked *JPH2* variants (Gly55Ala and Glu85Lys) (193, 211). Among genetic *JPH2* variants linked to cardiac disease and/or with predicted loss-of-function effects, some hotspots can be observed in TABLE 2. Variants in three consecutive residues (Tyr141, Gln142, and Val143) cause HCM or predicted LOF. There are five variants in the nine-amino acid peptide (Thr161-Glu169), four of which have been linked to HCM and three to LVOTO, suggesting that dysfunction of this domain might promote basal septal hypertrophy. Three variants between Ala181 and Ala189 cause cardiomyopathy (2 DCM and 1 HCM), suggesting a potential link to DCM-specific pathophysiology. While there appears to be some clustering, *JPH2* variants has been reported throughout the protein (FIGURE 16). It might be premature to derive structure-function relationships due to the limited availability of clinical data for variant carriers.

Cardiac arrhythmias have also been reported as part of the clinical phenotypes associated with *JPH2* variants. Atrial fibrillation (AF) was reported in a small, multigenerational pedigree of patients with HCM carrying the Glu169Lys variant (96, 187). AF was also documented in 9 of 26 HCM patients with the Thr161Lys variant (195). Interestingly, over 50% of patients with these same variants also exhibited cardiac conduction abnormalities. Various degrees of atrioventricular block and more distal conduction system deficits have also been reported in patients with the Glu85Lys, Ala405Ser, and Glu641Ter variants (96, 181, 193, 201). Finally, ventricular arrhythmias and sudden cardiac arrest/death have been reported in conjunction with three JPH2 variants: Ala189Thr (196), Ser241Arg (195), and Glu338Gly (199). Additional details about clinical manifestations in patients with *JPH2* variants are provided in TABLE 2.

#### 5.2.3. Functional consequences of JPH2 variants.

Well-established functional studies showing a deleterious effect of a genetic variant represent one of the main types of supporting evidence needed to classify variants as pathogenic. Studies of the in vitro and in vivo effects of *JPH2* variants on cardiac myocyte and heart function, respectively, have added new insights into the role of JPH2 in the heart. The first three *JPH2* variants identified in HCM patients were overexpressed in the H9C2 rat cardiomyoblast cell line to study their effects on cell size (180). The Tyr141His and Ser165Phe variants led to the induction of hypertrophic growth in this cellular model. In addition, there was an altered localization pattern with respect to the sarcoplasmic reticulum (SR) and the amplitude of intracellular Ca^2+^ transients was reduced in cells expressing each of these three *JPH2* variants (180). In a follow-up paper, Landstrom et al. (138) demonstrated that *JPH2* knockdown in atrial HL-1 cells caused cellular hypertrophy and led to activation of prohypertrophic markers such as ANF and BNP. However, this paper did not identify which prohypertrophic signaling pathways might be activated by JPH2 expression loss.

Quick et al. (202) developed a mouse model to study the Ala405Ser variant found in patients with HCM, septal hypertrophy, and LVOTO. Pseudo-knockin (PKI) mice with the Ala399Ser variant, equivalent to Ala405Ser in humans, were generated by crossing JPH2-Ala399Ser transgenic mice with inducible cardiac-specific *Jph2* knockdown mice. The offspring were dosed with tamoxifen to induce shRNA-mediated knockdown of total JPH2 levels to achieve cardiac protein levels similar to those in non-transgenic mouse hearts (202). Cardiac imaging protocols using echocardiography and cardiac magnetic resonance imaging revealed progressive LV and septal thickening reflective of the proband’s septal morphology. Histological analysis revealed cellular hallmarks of HCM. These studies revealed for the first time a causal link between a *JPH2* variant and HCM (202, 212).

As mentioned above, AF has been reported in HCM patients with the Glu169Lys variants in JPH2 (96, 187). Beavers et al. (96) performed programmed electrical stimulation studies that revealed that Glu169Lys-PKI mice had an elevated susceptibility to AF compared with WT-PKI mice or Ala399Ser-PKI mice. The Ala399Ser variant (Ala405Ser in humans) has been associated with HCM but not AF in patients (202). In isolated atrial myocytes from Glu169Lys-PKI mice, an increased incidence of spontaneous SR Ca^2+^ waves and SR Ca^2+^ release events was noted, suggestive of RyR2 hyperactivity (96). Coimmunoprecipitation experiments revealed that the Glu169Lys mutation reduces the binding of JPH2 to RyR2, thereby increasing activity of this SR Ca^2+^ release channel (96). It is interesting that the only other *JPH2* variant linked to AF thus far is Thr161Lys (195), suggesting that the molecular mechanism might be similar for both variants. Both variants are found in the flexible “joining domain” connecting the two sets of MORN domains. A small JPH2-derived peptide containing the Glu169 residue was able to reduce RyR2 channel activity, suggesting that JPH2 binding to RyR2 through this domain might be important for normal E-C coupling (96).

Finally, future studies may harness the power of new technologies like clustered regularly interspaced short palindromic repeats (CRISPR) genome editing in combination with human-induced pluripotent stem cell (hiPSC)-derived cardiomyocytes to decipher mechanisms by which genetic variants in *JPH2* cause cardiac disease (213, 214). While such studies have been performed on hiPSCs from patients with HCM (215), there is currently no such lines with cardiomyopathy-linked *JPH2* variants. On the other hand, Wu et al. (216) recently generated a *JPH2* biallelic knockout (KO) line of human embryonic stem cells using an episomal vector-based CRISPR/Cas9 system. This *JPH2*-KO hESC-line maintained stem cell-like morphology, pluripotency, had a normal karyotype, and could differentiate into all three germ layers in vivo. These and other human cell lines might be useful for future in vitro studies into the mechanisms by which *JPH2* loss-of-function variants cause HCM.

#### 5.2.4. Skeletal muscle myopathy linked to JPH2 variants.

While *JPH2* variants have been associated with cardiomyopathy and arrhythmias, a small number of studies have linked these variants to perturbed skeletal muscle function in vitro. Woo et al. (122) studied the effects of the Ser165Phe variant in JPH2 in mouse primary skeletal myotubes because RyR2 is also expressed in skeletal muscle cells. Overexpression of JPH2-Ser165Phe resulted in hypertrophy and increased resting Ca^2+^ levels, whereas the gain of E-C coupling and RyR1-mediated SR Ca^2+^ release were both reduced (122). Immunoprecipitation assays revealed that the Ser165Phe mutation impairs PKC-mediated phosphorylation resulting in impaired binding to the canonical-type transient receptor potential cation channel 3 (TRPC3) on the T-tubule membrane (122). Further studies revealed that since TRPC3 directly binds to and regulates RyR1, it was concluded that the Ser165Phe mutation promotes skeletal myocyte hypertrophy through impaired regulation of RyR1 function (122).

A subsequent study revealed that the Tyr141His variants in JPH2 also induces skeletal muscle hypertrophy, an increased resting Ca^2+^ level, and reduced E-C coupling gain (217). Unlike the Ser165Phe variant, however, the Tyr141His variants did not affect RyR1-mediated SR Ca^2+^ release nor PKC-mediated JPH2 phosphorylation (217). Rather, there was enhanced store-operated Ca^2+^ entry (SOCE) via Ca^2+^ release-activated calcium channel protein 1 (ORAI1), suggesting that the molecular mechanism is different for both mutations. Whereas these studies are certainly interesting, there is no clinical evidence for skeletal muscle myopathy, particularly not weakness, in probands hosting these or any other cardiomyopathy-linked JPH2 variant (180). Future studies in knockin mouse models might reveal whether these skeletal muscle phenotypes observed in cell lines can be replicated in vivo.

### 5.3. JPH3 Gene Repeats and Huntington Disease-Like 2

Inherited defects in *JPH3* were the first junctophilin gene disorder associated with a human disease, namely Huntington Disease-Like 2 (HDL2) (17). Like Huntington Disease, HDL2 usually presents in adulthood (during the fourth decade of life) with a relentless progressive triad of movement, psychiatric, and cognitive abnormalities, which lead to death within 10 to 20 yr (218). HDL2 is clinically indistinguishable from Huntington Disease. However, unlike Huntington Disease, HDL2 has been described exclusively in individuals with African ancestry. More than half of the individuals with HDL2 are from South Africa, while most of the remaining individuals reside in North and South America (219). In addition, HDL2 patients show movement disorders including eye motion abnormalities, increased Parkinsonism, chorea, hypokinesia (rigidity, bradykinesia), dysarthria, and hyperreflexia in the later stages of the disease. There is a strong correlation between the disease duration and progression of the motor and cognitive disorder (218). Dementia is a universal feature of HDL2. Depression, apathy, and irritability are the most common types of psychiatric manifestations (219).

The etiology of HDL2 is attributed to CAG/CTG repeat expansions on chromosome 16q24.2 (FIGURE 2), which is located on the sense strand in alternatively spliced exon 2 A of *JPH3* which is not part of the primary *JPH3* transcript (FIGURE 3) (220). Like other trinucleotide repeat disorders, also known as microsatellite expansion diseases, HDL2 is caused by a trinucleotide repeat expansion (CAG/CTG) that ranges normally form 6 to 28, whereas expansion to 40–59 repeats causes HDL2 (15). HDL2 is inherited in an autosomal-dominant manner. The length of the trinucleotide expansion has an inverse correlation with the age of disease onset, similar to Huntington Disease (219, 221). Of Black South African patients with symptoms of Huntington Disease who tested negative for Huntington (*HTT*) gene expansions, ∼35% will have expansions of the *JPH3* gene consistent with HDL2. In North Americans, ∼1% of patients with symptoms of Huntington Disease have *JPH3* expansions (222, 223).

There are currently three main hypothesis about the molecular pathogenesis of HDL2. First, the CTG repeat is transcribed and the RNA has toxic properties perhaps similar to myotonic dystrophy (224). Consistent with this theory, FISH and immunohistochemistry of brain samples from HDL2 patients revealed RNA foci within neurons. These RNA foci were not seen in HD patients but resembled those typically seen in patients with myotonic dystrophy (224). Furthermore, overexpression of the CUG repeat-containing alternatively spliced exon 2A resulted in cellular toxicity in vitro (224). Second, the repeat causes a loss of JPH3 protein, perhaps through sequestration of transcripts, before or after splicing, that are unavailable for translation (165). Third, a cryptic gene on the antisense encodes an expanded tract of polyglutamine residues, leading to polyglutamine toxicity perhaps similar to that in other polyglutamine disorders such as HD (165, 225). In the antisense strand, the repeat consists of a CAG repeat predicted to encode a polyglutamine tract within a cryptic unnamed gene. The antisense gene does appear to be transcribed, though whether it is transcribed when the repeat is expanded, and whether the transcript is translated, remain unknown (165, 226). One study stowed ubiquitinated intracellular inclusions that stain with 1C2 antibody that detects polyglutamine (polyQ) proteins in brains from HDL2 patients (225), supporting this hypothesis. On the other hand, another study did not detect expanded polyalanine or polyleucine peptides in brain samples from some HDL2 patients (165). Thus, in summary, several pathways may contribute to the pathogenesis of CAG/CTG repeat expansions in the *JPH3* gene, including CUG RNA toxicity, loss of JPH3 protein function, and polyQ protein toxicity.

### 5.4. Common Variants in JPH Genes

Genome-wide association studies (GWAS) identify associations between genetic regions (loci) and traits (i.e., diseases or clinical phenotypes) in an unbiased manner. Typical GWAS studies can identify common variants in a number of individuals, both with and without a common trait, across the entire genome, using genome-wide single nucleotide polymorphism (SNP) arrays. The *P* value indicates the significance of the difference in allele frequency between cases and controls, thus representing the probability that the allele is likely to be associated with the specific trait. The most commonly accepted threshold for a significant association is *P* < 5 × 10^−8^, which is based on performing a Bonferroni correction for all the independent common SNPs across the human genome (227). More recently, it has been suggested that the conventional threshold should be modified to take into account the increasing prevalence of low-frequency genetic variants in GWAS (228). SNPs near *JPH* genes have been associated with a wide range of phenotypes, but none reached the statistical threshold level and they have not been explored in sufficient depth to demonstrate *JPH* involvement. GWAS studies in larger populations and post-GWAS confirmatory studies are needed to determine whether the *JPH* gene products are involved in the aforementioned traits.

## 6. ACQUIRED DISEASES CAUSED BY ALTERED JUNCTOPHILIN FUNCTION

While inherited variants in *JPH* genes can cause various genetic diseases summarized in sect. 5, there is also a growing list of acquired diseases that are caused by altered levels or function of junctophilins. The following sections will summarize disease-relevant insights obtained from studies of human tissue samples, animal models of disease, and *Jph* knockout animals, among others.

### 6.1. Skeletal Muscle Disorders

#### 6.1.1. Skeletal myopathies.

Loss of JPH1 expression has been shown to reduce the number of intact JMCs/triads, to deform intact triads, and to reduce contractile force (141) in skeletal muscle, which can lead to myopathy, a clinical disorder characterized by muscle weakness with or without pain and/or inflammation. Abnormalities of muscle cell structure and metabolism lead to various patterns of weakness and dysfunction. Alterations in other proteins involved in triad formation and maintenance, including caveolin-3 (CAV3), amphiphysin-1 (BIN1), dysferlin (DYSF), myotubularin (MTM1), and striated muscle preferentially expressed protein kinase (SPEG), have also been linked to skeletal myopathy phenotypes in patients and rodent models (118, 132, 229).

A loss of JPH1 causes a reduced Ca^2+^-activated twitch tension as a result of JMC deficits and uncoupling of the voltage-gated Ca^2+^ channel and RyR1. Interestingly, mice heterozygous for the gain-of-function RyR1 variant Ile4895Thr, which is associated with central core disease in humans, develop a slowly progressive myopathy with skeletal muscle weakness and age-dependent formation of cores in their muscle fibers (230). Another RyR1 variant Tyr522Ser causes continuous Ca^2+^ leakage, high levels of reactive nitrogen and oxygen species, and the development of a myopathy characterized by decreased muscle performance and mitochondrial damage (231). Defects in other Ca^2+^ channel subunits including the voltage-gated Cav1.1 channel, calsequestrin 1 (CASQ1), stomal interaction molecule 1 (STIM1), and Ca^2+^ release-activated Ca^2+^ modulator 1 (ORAI1) have all been linked to myopathies (232).

Lengthening or eccentric contractions of skeletal muscle can lead to immediate and prolonged reductions in force-producing capacity (233). The early-stage force deficits have been attributed to an inability to stimulate SR Ca^2+^ release, i.e., E-C coupling failure. Corona et al. (234) showed that JPH1 was downregulated after skeletal muscle damage caused by eccentric contraction, a type of contraction in which the muscle elongates under tension due to an opposing force which is greater than the tissue-generated contractile force generated by the muscle. This loss of JPH1 coincided with the loss of E-C coupling, while the gradual restoration of JPH1 protein levels correlated with a recovery of E-C coupling and force generation. Further studies revealed that increased skeletal muscle contraction, as well as directly increasing cytosolic Ca^2+^ levels to supraphysiologic levels resulted in calpain-mediated proteolysis of JPH1 (235, 236).

#### 6.1.2. Muscular dystrophies.

Muscular dystrophies are a group of more than 30 genetic diseases characterized by progressive muscle weakness and age-dependent degeneration (237–239). Duchenne muscular dystrophy (DMD) is the most common form and primarily affects boys (240, 241). The absence of dystrophin causes muscle atrophy, increased fatty and immune infiltration, abnormal fiber distribution, enhanced oxidative stress, and E-C coupling deficits (241, 242). A recent proteomic study of skeletal muscle biopsies from DMD patients revealed a reduction in JPH1 protein levels, while calmodulin (CALM2) levels were increased (243). Similar findings were reported in the *mdx* mouse model of DMD, in which *Jph1* gene expression levels were found to be lower compared with C57 control mice (244). Cleavage of JPH1 occurs close to the COOH terminus, creating a 75-kDa diffusible fragment and a fixed 15- to 17-kDa fragment, with a loss of expression levels of the full-length protein (235). Interestingly, aberrant JPH1 proteolysis was seen in the *mdx* mouse model of muscular dystrophy, providing an association with the pathogenesis of a primary muscle disease (235). Endogenous µ-calpain (also known as calpain-1) was shown to proteolyze JPH1 at increased Ca^2+^ levels in human skeletal muscle cryosections and mouse tibialis anterior muscle (235). It is presently unknown whether other mechanisms such as altered transcriptional control, RNA processing, or translational control contribute to the loss of JPH1 in muscular dystrophies.

#### 6.1.3. Ischemic muscle injury.

Ischemia-reperfusion (I/R) injury is a condition that can be caused by prolonged blood flow disruption or acute compartment syndrome, followed by tissue reperfusion as a result of blood flow restoration or a repair procedure (245). In injured skeletal muscles from rats subjected to I/R injury, a 50% reduction in JPH1 protein levels was observed. It is well known that I/R injury leads to intracellular Ca^2+^ overload and calpain activation (10, 120, 246, 247), but the direct link to loss of JPH1 has not been studied. As indicated above, calpain-mediated proteolysis can produce shorter JPH1 fragments, the function of which is still unknown (235). Finally, it has been shown that exogenously applied nitric oxide by means of l-arginine (a nitric oxide precursor) decreased calpain-mediated proteolysis in tibialis anterior muscles subjected to eccentric contraction (236). *S*-nitrosylation of calpain was shown to partially prevent proteolysis of JPH1 in this model (236), suggesting that this might be an interesting therapeutic target for skeletal muscle injury.

### 6.2. Cardiomyopathies

#### 6.2.1. Hypertrophic cardiomyopathy.

Several studies have demonstrated that JPH2 protein levels are downregulated in various types of cardiomyopathy, a common type of chronic disease of the heart muscle (248). Landstrom et al. (138) reported reduced JPH2 expression levels in left ventricular septal tissue removed during surgical myectomy procedures for the treatment of obstructive HCM. In the Ras-transgenic mouse model of HCM, *Jph2* mRNA levels were downregulated by 60% and JPH2 protein levels were decreased by 40% (111). These mice were previously shown to exhibit a reduced gain of E-C coupling, since the L-type Ca^2+^ current was unaltered but the SR Ca^2+^ transient was reduced (249). Similar findings were obtained in rats subjected to ascending aortic stenosis surgery, which led to the development of compensated hypertrophic cardiomyopathy (250). The reduction in *Jph2* mRNA and JPH2 protein levels was accompanied by an impaired gain in E-C coupling and dyssynchrony of SR Ca^2+^ release (250). Partial silencing of *JPH2* expression in HL-1 cells using a small interfering RNA probe targeting murine *Jph2* mRNA (shJPH2) resulted in myocyte hypertrophy and increased expression of known markers of cardiac hypertrophy (138). Knockdown of JPH2 expression led to depressed maximal Ca^2+^ transient amplitudes that were insensitive to L-type Ca^2+^ channel activation, consistent with the findings obtained in rodent ventricular myocytes (138). These findings suggest a causal link between the loss of JPH2 expression levels, cardiomyopathy, and alterations in SR Ca^2+^ handling. Therefore, normalizing JPH2 protein levels might represent a promising therapeutic strategy for the treatment of HCM, which might lead to normalized E-C coupling.

#### 6.2.2. Dilated cardiomyopathy.

A reduction in JPH2 protein levels has also been reported in the muscle-specific LIM protein (MLP) knockout mouse model of DCM (251, 252). These mice, however, did not exhibit a change in *Jph2* mRNA levels, suggesting that the expression of JPH2 might be regulated at the posttranscriptional level in the MLP-KO mice (111). The functional consequence of the loss of JPH2 levels in MLP-KO mice was a reduced SR Ca^2+^ transient, consistent with impaired E-C coupling (251).

Reduced JPH2 expression levels have also been reported in a mouse model of lipotoxic cardiomyopathy. Male C57BL/6 mice that were fed a 60% high-fat diet for 12 wk developed myocardial hypertrophy, fibrosis, reduced coronary reserve, and suppressed cardiac function (253). The reduced JPH2 expression levels could be reversed by swimming exercise and silencing of 3-hydroxy-3-methylglutaryl-CoA synthase 2 (HMGCS2), suggesting that the cardiomyopathy development itself led to loss of JPH2.

Duchenne muscular dystrophy (DMD), an X-linked disease characterized by striated muscle dysfunction, leads to cardiomyopathy in the majority of patients. Prins et al. (254) found that reduced JPH2 protein levels correlated with increases in the total microtubule content in the *mdx* mouse model of DMD. Interestingly, colchicine-mediated microtubule depolymerization normalized JPH2 levels, restored T-tubule organization, and reduced the frequency of Ca^2+^ sparks. These studies suggest that loss of JPH2 contributes to SR Ca^2+^-handling deficits in the hearts of mdx mice (239–242, 254, 255). Together, these studies suggest that restoring JPH2 expression levels might be a promising strategy for the treatment of DCM, although preclinical validation studies are required to confirm this.

#### 6.2.3. Left ventricular noncompaction cardiomyopathy.

Left ventricular noncompaction cardiomyopathy (LVNC) is a rare genetic condition of the heart where the left ventricular wall is not compacted. This leads to endomyocardial trabeculations of the heart, which is typically caused by an arrest of normal maturation of the myocardium (185). It has been shown that subcellular redistribution of JPH2 without changes in its protein levels can contribute to loss of E-C coupling in a mouse model of LVNC (256). Proteomics-based studies of human heart tissues obtained from transplant patients identified sorbin and SH3 domain-containing protein 2 (SORB2) as a protein specifically elevated in LVNC as compared with HCM and ARVC (256). SORB2 regulates cytoskeleton dynamics, and overexpression of SORB2 leads to β-tubulin polymerization. On the other hand, knockout of SORB2 disrupts the structural integrity of the intercalated disk and manifests in mice as features of arrhythmogenic cardiomyopathy (257). Further research is required to investigate the potential interaction between SORB2 and JPH2 in the context of cardiomyopathy development.

### 6.3. Pulmonary Hypertension

Pulmonary hypertension (PH) is a disorder defined by an increase in mean pulmonary arterial pressure of ≥25 mmHg at rest as assessed by right heart catheterization (258). This disease can occur in the context of various pulmonary, vascular, and cardiac conditions, including congenital cardiomyopathies. In a rat model of PH induced with a single injection of monocrotaline, right ventricular cardiomyocytes exhibited severe loss and disorganization of T-tubules (158). This was also associated with blunted and dyssynchronous SR Ca^2+^ release. Interestingly, sildenafil, a phosphodiesterase 5 inhibitor, prevented and partially reversed ultrastructural remodeling of T-tubules and SR Ca^2+^ handling (158). Prins et al. (259) showed that JPH2 protein levels are downregulated in the right ventricle of rats with PH. In this model, colchicine also reduced microtubule density, restored JPH2 expression, and improved T-tubule organization in right ventricular myocytes. These findings suggest a functional interaction between JPH2 and the microtubules in right ventricular myocytes.

### 6.4. Heart Failure

#### 6.4.1. JPH2 levels in heart failure.

Heart failure (HF) is a clinical syndrome characterized by a reduced ability of the heart to meet the metabolic demands of the body. The syndrome of HF commonly overlaps other cardiovascular diseases, such as coronary artery disease, hypertension, valvular disease, and primary myocardial disease (260). It is well established that altered intracellular Ca^2+^ homeostasis plays a key role in the pathophysiology of human heart failure (10, 261). Protein expression levels of JPH2 were found to be reduced in patients with ischemic heart failure (262) and a rat model of decompensated heart failure (250). The reduction of JPH2 downregulation was found to correlate with the severity of systolic heart failure (154).

Germline knockout of *Jph2* in mice led to the development of embryonic heart failure as early as embryonic day E9.5 (2). Since these global *Jph2* knockouts all died by E11.5, van Oort et al. (6) developed an inducible mouse model to downregulate JPH2 levels in adult animals. Downregulation of JPH2 levels in cardiac myocytes using a small hairpin RNA (shRNA) leads to a rapid development of systolic heart failure and pathological remodeling. These mice demonstrated grossly enlarged hearts with dilated ventricles and reduced systolic function on echocardiograms, confirming that loss of JPH2 is sufficient to cause HF development (6). On the other hand, restoring JPH2 levels using adeno-associated virus type 9 (AAV9) improved cardiac function in mice with early stage HF caused by pressure overload (263). The restoration of JPH2 levels prevented the loss of T-tubules and normal SR Ca^2+^ handling deficits associated with contractile failure in this mouse model. Importantly, transgenic overexpression of JPH2 did not affect baseline cardiac function, whereas it did provide significant protective benefits after pressure overload in mice (264). These findings suggest that JPH2 might be an attractive therapeutic target for treating pathological cardiac remodeling during HF (263).

#### 6.4.2. Functional effects of reduced JPH2 levels in failing hearts.

At the cellular level, acute loss of JPH2 expression causes a loss of E-C coupling gain due to a rapid deterioration of JMCs and an increased variability in spacing between the juxtaposed membranes within dyads (6). Reorganization of the T-tubule network was found to occur early during remodeling after pressure-overload induced hypertrophy in rats and became worse as moderate and severe HF ensued during disease progression (154). A reduction of the density of T-tubules is a common finding in other animal models including a canine model of tachycardia-induced HF (265), a mouse model of myocardial infarction (155), and rat model of pressure-overload induced HF (266). Wagner et al. (155) used superresolution STED microscopy to characterize individual T-tubules after myocardial infarction. T-tubule morphology and associated proteins including JPH2 were found to be altered early during HF development, leading to T-tubule network reorganization (155). With the downregulation of JPH2, there is a decrease in transverse T-tubular structures and an increase in axial (longitudinal) structures, similar to changes in the development of perinatal T-tubule with loss of JPH2 (148). The loss of JPHs also leads to physical uncoupling of T-tubules from the sarcoplasmic reticulum in the failing heart (145, 267). Interestingly, Lyon et al. (268) reported a reduction in JPH2 protein expression and a loss of T-tubule density in a postinfarction rat model of HF. Interestingly, AAV9-mediated overexpression of SERCA2a improved T-tubule density, despite a lack of normalization of JPH2 levels. However, the expression levels of other proteins important for T-tubule structure, including BIN1 and TCAP, did normalize, suggesting that normalization of JPH2 may not be a prerequisite for T-tubule restoration in this model (268).

The knockdown of JPH2 protein levels in cardiac myocytes was found to enhance spontaneous openings of the RyR2 channel and promote spontaneous SR Ca^2+^ leak (6). In mice with myocardial infarction, loss of JPH2 was associated with dyssynchrony of the release of Ca^2+^ from JMCs across the Z-line in cardiac myocytes (155, 269). Similar findings were obtained in rat myocytes in which JPH2 was knocked down, since dyssynchrony of RyR2 activation and SR Ca^2+^ release was reported (266). Disruption of the JMC architecture due to JPH2 loss causes RyR2 to relocalize to non-JMC areas, leading to “orphan” RyR2 (155, 270). The observation that JPH2 directly binds to RyR2 and reduces channel gating suggests that loss of JPH2 expression in failing cardiomyocytes may directly be responsible for defects in SR Ca^2+^ release (96). Computational modeling of heterogeneous changes in T-tubule components and orphaning of RyR2 channels revealed evidence for delayed SR Ca^2+^ release, blunted activation of the systolic Ca^2+^ transient, and persistent diastolic RyR2 Ca^2+^ leak, all consistent with experimental findings (155). On the other hand, increasing JPH2 expression in *Jph2*-knockdown mouse myocytes increased the Ca^2+^ transient amplitude and synchronization of SR Ca^2+^ release (262). In addition, it has been shown that *Jph2* knockdown in mouse ventricular myocytes led to a reduction in Na^+^/Ca^2+^-exchanger (NCX) activity despite unaltered NCX protein expression levels (271). Superresolution microscopy revealed that loss of JPH2 led to a reduced overlap between RyR2 and NCX, suggesting that increased SR Ca^2+^ leak can be caused by a combination of enhanced RyR2 channel activity and reduced junctional NCX activity (271).

#### 6.4.3. Mechanisms underlying loss of JPH2 in heart failure.

Several molecular mechanisms have been shown to contribute to downregulation of JPH2 expression levels in failing hearts (28, 272). First, the mouse, rat, and human homologues of JPH2 are regulated by microRNA-24 (miR-24), which is upregulated in HF (267, 273). Luciferase assays revealed that miR-24 binds to two redundant binding sites in the 3′-untranslated region of the *Jph2* gene (273). Overexpression of miR-24 was shown to reduce the number of T-tubule-SR junctions, decrease E-C coupling gain, and promote SR Ca^2+^ release dyssynchrony (273). In vivo silencing of miR-24 using an antagomir in a mouse model of pressure overload led to preservation of the T-tubule architecture and normalization of Ca^2+^ handling (274). While suppression of miR-24 did not ameliorate cardiac hypertrophy, it did prevent the progression to decompensated HF (274). On the other hand, Qian et al. (275) demonstrated that miR-24 is downregulated in the ischemic border zone of the murine left ventricle following myocardial infarction. In their studies, overexpression of a miR-24 mimic inhibited cardiomyocyte apoptosis and reduced cardiac dysfunction, while inhibition of miR-24 promoted apoptosis (275). Therefore, targeting this miR-24 pathway may not be suitable for therapeutic targeting of JPH2 in the context of heart failure treatment.

Second, mislocalization of JPH2 as a result of an increased density of microtubules has also been observed in hypertrophied and failing mouse hearts (276). Microtubules, which are ubiquitous cytoskeletal filaments formed by polymerized α- and β-tubulin dimers, regulate a wide range of cellular processes, including maintenance of cell shape and intracellular protein transport. In human hypertrophied and failing myocardium, tubulin expression levels were found to be increased (277). Similarly, in a murine model of pressure-overloaded hypertrophy, microtubule densification correlated with T-tubular remodeling and E-C coupling loss (276). Furthermore, treatment of myocytes with colchicine, a microtubule destabilizer, disrupted T-tubular remodeling as well as JPH2 relocalization, which also normalized E-C coupling (276). The same mechanism has been reported in muscular dystrophy-associated cardiomyopathy (254) and in failing right ventricles as a result of pulmonary hypertension (259).

Third, proteolytic cleavage has been identified as a key mechanism of JPH2 downregulation. Endogenous µ-calpain (also known as calpain-1) was shown to proteolyze JPH2 and JPH1 at Ca^2+^ concentrations of 0.5–5 µM (235). It was shown that prolonged exposure to 0.5 mM of Ca^2+^ concentration (a very high physiological concentration) or ischemia-reperfusion injury to the rat heart led to proteolysis of JPH2 (235). Sustained elevation of intracellular Ca^2+^ levels led to autocatalytic activation of μ-calpain, which in turn cleaved JPH2 into diffusible and fixed fragments. In a mouse model of inducible Gα_q_ activity, JPH2 cleavage was observed, which was reversed by treatment with a calpain inhibitor (278). Interestingly, calpain-1 levels were found to be reduced in the inducible Gα_q_ mice with increased JPH2 cleavage, suggesting that this might be a compensatory response. On the other hand, in patients with ischemic and dilated cardiomyopathy, calpain-1 expression levels were found to be increased, while JPH2 levels were reduced (248, 279). Calpain activity levels were also found to be increased in three different mouse models of heart failure (transverse aortic constriction, myocardial infarction, and isoproterenol minipumps) (248). Pharmacological inhibition of calpain prevented HF development and T-tubule disorganization in each of these models. In contrast, overexpression of calpain-1 led to rapid onset HF and death in mice. Pretreatment with calpain inhibitor PD150606 prevented the proteolytic cleavage of JPH2 following ischemia-reperfusion of isolated mouse hearts (280). In addition, lipotoxicity as a result of a high fat diet was shown to cause cardiac injury associated with increased calpain activity (281). Genetic knockdown of calpain-1 and pharmacological inhibition of calpain prevented endoplasmic reticulum stress, apoptosis, and JPH2 cleavage in this model. Proteolysis of JPH2 can also be caused by matrix metalloproteinase-2 (MMP-2), a Zn^2+^- and Ca^2+^-dependent protease that is activated by oxidative stress. JPH2 cleavage was observed in isolated, perfused rat hearts subjected to ischemia-reperfusion (I/R) injury (282). Addition of MMP-2 inhibitor ARP-100 prevented JPH2 proteolysis, contractile dysfunction, and damage to the dyads. MMP was found to bind to JPH2 and to cleave JPH2 between the MORN repeats and within the divergent domain (282).

#### 6.4.4. JPH2 cleavage fragments.

**FIGURE 17.**
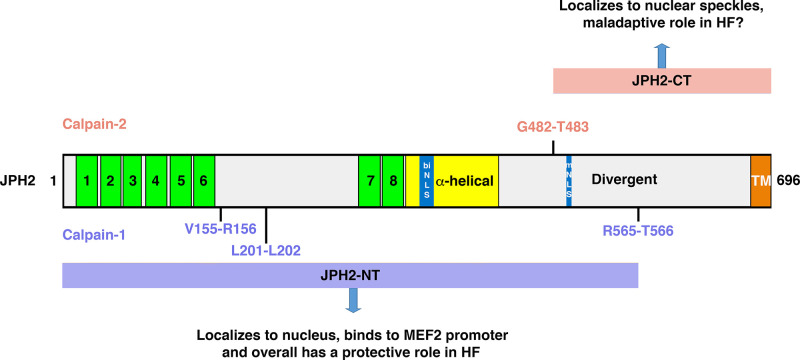
Proteolytic pathways of junctophilin-2 in diseased hearts. Schematic showing the protein structure of junctophilin-2 (JPH2) in which major functional domains are highlighted: membrane occupation and recognition nexus (MORN) domains in green, alpha-helical domain in yellow, and transmembrane domain (TM) in orange. Calpain-1 cleavage sites are shown in blue, and calpain-2 cleavage sites are shown in pink. Resulting proteolytic fragments are shown, and their subcellular functions are indicated. biNLS, biphasic nuclear localization signal; mNLS, monopartite nuclear localization signal; HF, heart failure; MEF2, myocyte enhancer factor 2.

Proteomics analysis revealed a calpain cleavage site in JPH2 at L201-L202 (278) (FIGURE 17). In vitro ischemia-reperfusion injury in mouse hearts also led to JPH2 cleavage, which was prevented by treatment with a calpain inhibitor or overexpression of calpastatin, an endogenous calpain inhibiting protein (262). In this study, several calpain cleavage sites were identified and mutagenesis defined the COOH-terminal region as the predominant calpain cleavage site (262). Cleavage of the COOH-terminal site (Arg565-Thr566) was suggested to be a prerequisite for proteolysis of the NH_2_-terminal sites (Val155-Arg156 and Leu201-Leu202) (262). Overexpression of several of these NH_2_- and COOH-terminal JPH2 peptides in myocytes isolated from *Jph2*-knockdown mice failed to normalize SR Ca^2+^ transients (262) unlike full-length JPH2 (6), suggesting that the proteolytic JPH2 peptides might not be involved in regulating E-C coupling (279).

Using several domain-specific antibodies, Lahiri et al. (283) identified a novel COOH-terminal JPH2 proteolytic peptide with an apparent molecular mass of 25 kDa. The peptide cleavage site was identified as Gly482-Thr483 (in the mouse JPH2 sequence) using truncation and site-directed mutagenesis (283). It was shown that this COOH-terminal peptide (CTP) was cleaved by calpain-2 (m-calpain), unlike the aforementioned peptides that are cleaved by calpain-1. Interestingly, the JPH2-CTP was shown to translocate into the nucleus using a monopartite nuclear localization signal (mNLS) in ventricular myocytes isolated from mice suffering from pressure-overload induced HF (see FIGURE 3). Prevention of this nuclear translocation of JPH2-CTP protected cardiomyocytes from developing isoproterenol-induced hypertrophy in vitro (283). In another study, Guo et al. (284) found an NH_2_-terminal peptide (NTP) cleaved at Arg585-Thr566 that also translocates into the nucleus. They found that nuclear import required the mNLS but not the bipartite NLS (bNLS) within the JPH2-NTP (see FIGURE 3) (284). On the other hand, the bNLS mediates binding to genomic DNA within the nucleus. Overexpression of JPH2-NTP led to repression of myocyte enhancer factor 2 (MEF2)-mediated transcription by competition with the MEF2 response element (284). Overexpression of JPH2-NTP was shown to attenuate pathological remodeling in response to cardiac stress, suggesting that this peptide might be part of a self-protective mechanisms that counters pathological transcriptional remodeling. Thus the NTP cleaved from JPH2 can act as a transcriptional regulator that is transferred into the nucleus and binds to promoter regions of target genes (284).

### 6.5. Cardiac Arrhythmias

#### 6.5.1. Atrial fibrillation.

Alterations in JPH2 expression levels can also lead to cardiac arrhythmias, which are, by definition, abnormal rhythms of the heart. Atrial fibrillation (AF) is the most common sustained cardiac arrhythmia that affects over 6,000,000 Americans (285, 286). Patients with early stage, paroxysmal AF had reduced JPH2 levels per RyR2 channel compared with patients in sinus rhythm (96). Complimentary studies in JPH2-overexpression, nontransgenic, and JPH2 knockdown mice revealed that atrial JPH2 levels correlated negatively with the incidence of pacing-induced AF (96). These findings suggest that binding of JPH2 to RyR2 channel complexes exerts a stabilizing function that helps prevent aberrant SR Ca^2+^ release events (96, 287). Consistent with this observation is the finding that the Glu169Lys variant in JPH2, identified in patients with HCM and AF, reduces binding to RyR2, hereby increasing abnormal SR Ca^2+^ release events and the incidence of AF in Glu169Lys mutant mice (96). Conversely, addition of a small 25-aa JPH2 peptide corresponding to the region surrounding residue Glu169 stabilized RyR2 in cardiomyocytes isolated from JPH2-deficient mice. Ni et al. (288) demonstrated that atrial-specific knockdown of *Jph2* using adeno-associated viral (AAV) vectors was associated with abnormal SR Ca^2+^ handling in atrial myocytes. Thus, arrhythmogenic SR Ca^2+^ leak and arrhythmias may be triggered by loss of the RyR2-stabilizing interaction of JPH2 (145).

#### 6.5.2. Premature ventricular contraction-induced cardiomyopathy.

Frequent premature ventricular contractions (PVCs) have been associated with an increased risk of sudden cardiac death. Wang et al. (289) developed a canine model of frequent PVCs and showed that despite LV contractile dysfunction, there were no detectable structural abnormalities at the macroscopic level. However, there was clear evidence for JMC remodeling with disarray of Cav1.2 distribution. In a follow-up study, the same group showed that Cav1.2 is downregulated and misplaced from T-tubules and that JPH2 is also downregulated (97). Consistent with prior results (271), loss of JPH2 was associated with loss of Na^+^/Ca^2+^-exchanger from the dyads (97, 290). These interesting results suggest that normalizing JPH2 levels might represent a new therapeutic approach for the treatment of cardiomyopathy associated with frequent PVCs.

## 7. SUMMARY AND CONCLUSIONS

There is growing scientific evidence that junctophilin proteins play various cellular functions in excitable cell types. They play vital roles in membrane tethering and ion channel regulation within junctional membrane complexes (JMCs) of skeletal muscle, cardiac muscle, and neuronal cells. While JPH1 and JPH2 are critical for JMC biogenesis in skeletal and cardiac striated muscles, respectively, JPH2 also plays a role in arterial smooth muscle cells. Additionally, the roles of JPH3 and JPH4 are increasingly being elucidated in the soma and dendrites of central and peripheral neurons.

The unique structural domain organization of JPH proteins contributes to their abilities to precisely control functional activity in electrically active cells. Evolutionary conservation studies highlight the importance of the eight membrane occupancy and recognition nexus (MORN) domains in JPH isoforms, which exhibit important structural differences from related MORN domains in other protein families. While the endoplasmic/sarcoplasmic reticulum (ER/SR) membrane insertion pathways for tail-anchored proteins are well defined in yeast, little is known about subcellular processing of JPH isoforms in mammalian cells. Superresolution imaging studies have, however, revealed evidence for subcellular clustering of JPH1 and JPH2 within ER/SR organelles in striated muscle cells. JPH1/JPH2 proteins can form dimers and interact with ryanodine receptor (RyR2) on the ER/SR and voltage-gated L-type Ca^2+^ channels and Ca^2+^-activated potassium channels (i.e., SK and BK channels) on the plasma membrane.

Genetic variation in *JPH* genes has been linked to a variety of inherited striated muscle and neuronal disorders. Inherited *JPH1* variants act as a disease-modifier in Charcot-Marie-Tooth disease caused by variants in ganglioside-induced differentiation-associated protein 1 (*GDAP1*). Genetic variants in *JPH2* cause hypertrophic and dilated cardiomyopathy and in some cases also skeletal muscle myopathy. Trinucleotide repeat expansions in the *JPH3* gene cause a neurodegenerative disease known as Huntington Disease-Like 2 (HDL-2). Finally, genome-wide association studies (GWAS) have identified associations between genetic loci and various traits, although it remains to be established whether any of those disease phenotypes are directly caused by changes in *JPH* gene expression.

Acquired changes in JPH protein expression levels or proteostasis can contribute to the initiation and maintenance of disease pathogenesis in the striated muscle and neurons. For example, loss of JPH1 expression levels has been linked to skeletal myopathies and dystrophies. Reduced JPH2 protein expression has been observed and causally linked to various inherited cardiomyopathies, pulmonary hypertension, and heart failure. JPH2 levels can be reduced due to microRNA-mediated gene silencing, mislocalization due to microtubule densification, and proteolytic cleavage due to increased activity of calpains and matrix metalloproteinase-2. Reduced JPH2 expression levels and functional activity may also contribute to atrial fibrillation and premature ventricular contraction-induced cardiomyopathy. Studies in patient-derived inducible pluripotent stem cells and genetic animal models will continue to provide a deeper mechanistic understanding of *JPH2* gene regulation and functional activity in excitable cell types and their contributions to disease development.

Finally, given the important roles of JPH isoforms in various cell types and the direct association of JPH dysfunction and human diseases, correcting JPH protein levels or functional activities have emerged as a promising therapeutic target for various skeletal muscle, cardiac muscle, and neuronal diseases. Further investigations that span the translational gap from early to clinical studies are timely now to determine whether two decades of basic and clinical research can be converted into new therapeutic options for patients.

## GRANTS

X.H.T.W. is supported by grants from the National Institutes of Health (R01-HL091947, R01-HL117641, R01-HL134824, and R01-HL147108) and the Juanita P. Quigley Endowed Chair in Cardiology. S.E.L. is supported by Deutsche Forschungsgemeinschaft through SFB1190 (Project P03) and under Germany’s Excellence Strategy (EXC2067/1-390729940). S.E.L. is an investigator of DZHK (German Centre for Cardiovascular Research).

## DISCLOSURES

No conflicts of interest, financial or otherwise, are declared by the authors.

## AUTHOR CONTRIBUTIONS

S.E.L. and X.H.T.W. conceived and designed research; S.E.L. and X.H.T.W. analyzed data; S.E.L. and X.H.T.W. interpreted results of experiments; S.E.L. and X.H.T.W. prepared figures; S.E.L. and X.H.T.W. drafted manuscript; S.E.L. and X.H.T.W. edited and revised manuscript; S.E.L. and X.H.T.W. approved final version of manuscript.
